# Gene Expression Profiling in Preterm Infants: New Aspects of Bronchopulmonary Dysplasia Development 

**DOI:** 10.1371/journal.pone.0078585

**Published:** 2013-10-23

**Authors:** Jacek J. Pietrzyk, Przemko Kwinta, Embjorg J. Wollen, Mirosław Bik-Multanowski, Anna Madetko-Talowska, Clara-Cecilie Günther, Mateusz Jagła, Tomasz Tomasik, Ola D. Saugstad

**Affiliations:** 1 Department of Pediatrics, Jagiellonian University, Krakow, Poland; 2 Department of Pediatric Research, University of Oslo, Oslo, Norway; 3 Department of Medical Genetics, Jagiellonian University, Krakow, Poland; 4 Norwegian Computing Center, Oslo, Norway; Ludwig-Maximilians-University Munich, Germany

## Abstract

**Rationale:**

Bronchopulmonary dysplasia is one of the most serious complications observed in premature infants. Thanks to microarray technique, expression of nearly all human genes can be reliably evaluated.

**Objective:**

To compare whole genome expression in the first month of life in groups of infants with and without bronchopulmonary dysplasia.

**Methods:**

111 newborns were included in the study. The mean birth weight was 1029g (SD:290), and the mean gestational age was 27.8 weeks (SD:2.5). Blood samples were drawn from the study participants on the 5th, 14th and 28th day of life. The mRNA samples were evaluated for gene expression with the use of GeneChip® Human Gene 1.0 ST microarrays. The infants were divided into two groups: bronchopulmonary dysplasia (n=68) and control (n=43).

**Results:**

Overall 2086 genes were differentially expressed on the day 5, only 324 on the day 14 and 3498 on the day 28. Based on pathway enrichment analysis we found that the cell cycle pathway was up-regulated in the bronchopulmonary dysplasia group. The activation of this pathway does not seem to be related with the maturity of the infant. Four pathways related to inflammatory response were continuously on the 5^th^, 14^th^ and 28^th^ day of life down-regulated in the bronchopulmonary dysplasia group. However, the expression of genes depended on both factors: immaturity and disease severity. The most significantly down-regulated pathway was the T cell receptor signaling pathway.

**Conclusion:**

The results of the whole genome expression study revealed alteration of the expression of nearly 10% of the genome in bronchopulmonary dysplasia patients.

## Introduction

Bronchopulmonary dysplasia (BPD) is a chronic lung disease associated with premature birth and characterized by early lung injury [1]. The current consensus is that BPD is a complex disease, and its pathogenesis depends on the interaction of a susceptible host with a multitude of environmental risk factors. The disease is characterized by disturbed alveologenesis. The many factors that influence alveologenesis include growth factors, cytokines and other substances that may act as ligands, receptors, signaling molecules and transcription factors, and the proteins that are the products of cell activity, such as enzymes participating in matrix reconstruction, retinoids and elastin [2–4]. 

Several experimental trials indicate that growth factors, especially those associated with vascularization (VEGF-Vascular endothelial growth factor), are closely related to the morphological changes in the respiratory tract of children with BPD [3,5–8]. Researchers are also investigating inflammatory mediators, such as Tumor Necrosis Factor α (TNF-α), Interleukin-1β (IL-1β) Interleukin-6 (IL-6), Interleukin-8 (IL-8) and Interleukin-10 (IL-10) [9–11]. Atypical pathogens, especially *Ureaplasma* spp., are believed to play a particular role in the inflammatory reaction leading to BPD [12,13]. 

It is generally agreed that respiratory support in VLBW infants must be conducted in such a way as to circumvent damage caused by pressure, volume or oxygen. Results of a meta-analysis conducted by Stevens et al. demonstrate that extubation after early surfactant therapy and subsequent respiratory assistance with nasal continuous positive airway pressure results in a lower incidence of BPD compared with selective surfactant therapy and subsequent mechanical ventilation [14]. Other authors have presented similar observations favoring less invasive methods of respiratory assistance and lower ventilation values [15,16]. Oxygen therapy and the subsequent action of its derivates (free radicals) has been proven to increase the incidence of BPD [17,18]. To prevent such complications, practical guidelines recommending lower blood oxygen saturation values for preterm babies have been introduced [19,20].

Genetic foundations for the development of BPD are implicated in twin studies, which reveal highly significant concordance rates for BPD: 3.69-fold in monozygotic and 1.4-fold in dizygotic twins [21]. Introduction of the microarray technique into clinical studies was one of the most important breakthroughs responsible for the dramatic progress in the field of human genetics during the last decade. The use of microarrays has given a new opportunity for studying even 20 000 human genes in a single experiment. The scope of potential applications of the microarrays is very broad, combining research and clinical medicine. The greatest advantage of this method is that it enables assessment of a great number of genetic factors (practically all human gene expression) although only a small amount of blood is necessary for testing, which is very important in preterm neonates.

To explore the pathogenesis of BPD, we carried out genome wide transcriptional profiling of RNA extracted from peripheral blood mononuclear cells of BPD subjects and non-BPD controls followed by pathway enrichment analysis in an attempt to identify biological pathways that were preferentially associated with BPD.

## Methods

A prospective study was conducted between September 1, 2008 and November 30, 2010. The entry criteria were (a) preterm birth < 32 weeks gestational age, (b) birthweight ≤1500g, (c) the need for respiratory support. All patients were outborn in local hospitals and transported to the Polish American Children’s Hospital which is a tertiary care unit for the region. 

The majority of patients are referred from first-level neonatal care hospitals, which provide mainly for rural areas. Detailed perinatal history (birth weight, gestational age, Apgar score at 1 and 5 minutes after birth) and history of treatment in the referral hospital (mechanical ventilation, oxygen therapy, surfactant treatment, diagnoses) were taken on admission. Maternal fever/infection was used as surrogate for “clinical chorioamnionitis”. Data on histological chorioamnionitis were unavailable in most cases. Ureaplasma infection was defined as positive tracheal aspirate culture for *Ureaplasma urealythicum*. The study was approved by the Ethics Committee of the Jagiellonian University, Faculty of Medicine (KBET 27/B/2007). Written informed consent was obtained from all participants involved in the study.

### Microarray analysis

After obtaining written informed consent from the parents, blood samples (0.3 ml) were drawn from all the study participants on the 5th, 14th and 28th day of life (DOL) for the assessment of whole genome expression in peripheral blood leukocytes. Subsequently, Ficoll isopaque gradient centrifugation (30 min, 2100 rpm, RT), and two times wash in 1x PBS (12 min, 1600 rpm, 40C), and finally RiboPure Blood Kit (Ambion, Life Technologies, Carlsbad, USA) were used for total RNA extraction.RNA concentration was measured with the use of NanoDrop Spectrophotometer (NanoDrop ND-1000; Thermoscientific, Waltham, USA), and RNA quality was determined by 2100 Bioanalyzer (Agilent, Santa Clara, USA). 

100ng of total RNA were used for the single microarray experiment. GeneChip Human Gene 1.0 ST Arrays (Affymetrix, Santa Clara, USA) were used. Whole transcript microarray experiment was performed according to the manufacturer’s protocol (GeneChip Whole Transcript sense Target Labeling Assay Manual, Version 4). The Affymetrix GeneChip Whole Transcript sense Target Labeling Assay is designed to generate amplified and biotinylated sense-strand DNA targets from the entire expressed genome. The protocol is optimized for use with the GeneChip Sense Target Arrays, where the probes are distributed throughout the entire length of each transcript. Details of microarray experiment were presented in online supplemental file – Appendix S1. 

### Real-time PCR validation

To validate the results obtained by the microarray analysis real-time PCR technique was used. A total of 30 cDNA samples (15 patients with BPD and 15 controls) remaining after microarray analysis, were used for the validation procedure. Samples were randomly selected from the studied groups. 

From each sample 100ng of cDNA was used for the single TaqMan Gene Expression Assay. Amplification reaction was performed with the use of TaqMan Universal PCR Master Mix and appropriate TaqMan probes (Life Technologies, Carlsbad, USA). Each sample was analyzed in duplicate. 

Average between the expression of endogenous controls (housekeeping genes: GAPDH and Actin-B) was used for determination of the relative expression levels with the use of∆∆Ct calculation.For the validation procedure 14 genes were randomly selected from the group of genes differentially expressed in the microarray experiment (fold change greater or equal to +/-1.5) and from the genes without significant differences in expression.

Following TaqMan Gene Expression Assays were used: Hs00900055_m1 (VEGF gene), Hs00952786_m1 (AK5 gene), Hs00217864_m1 (OLAH gene), Hs01030384_m1 (ILR2 gene), Hs00187022_m1 (ADAM23 gene), Hs00736937_m1 (DAAM2 gene), Hs00360669_m1 (CD177 gene), Hs00255338_m1 (KLRC4 gene), Hs00171191_m1 (FBN1 gene), Hs00539582_s1 (LRRN3 gene), Hs00196254_m1 (NELL2 gene), Hs00924296_m1 (MPO gene), Hs00541549_m1 (ABCA13 gene), Hs00197437_m1 (OLFM4 gene),GAPDH (Hs02758991_g1) and Actin-B (Hs99999903_m1); (Life Technologies, Carlsbad, USA). 

### Outcome variables

Scheduled examinations were performed on all patients. The procedures of oxygen therapy in the examined group were consistent throughout the study with the goal of maintaining hemoglobin saturation between 88 and 95%. BPD severity was graded according to criteria proposed by Jobe and Bancalari [22]. Four cohorts of children were identified based on examination at age 36 weeks post conception or on discharge. Mild BPD was defined as a need for supplemental oxygen for > or =28 days but not at 36 weeks' postmenstrual age (PMA) or discharge; moderate BPD as a need for supplemental oxygen for > or =28 days plus treatment with <30% oxygen at 36 weeks' PMA, and severe BPD as a need for supplemental oxygen for > or =28 days plus > or =30% oxygen supplementation and/or positive pressure at 36 weeks' PMA. Infants treated with oxygen > 21% and/or positive pressure for nonrespiratory disease (e.g., central apnea or diaphragmatic paralysis) were not included in the BPD group unless they also developed parenchymal lung disease and exhibited clinical features of respiratory distress. Treatment with oxygen > 21% and/or positive pressure at 36-week postmenstrual age (PMA) due to an "acute" event was not a criterion for BPD. The evaluation of oxygen requirement was performed by a staff member blinded to molecular studies results.

### Statistical analysis

The R package, *sizepower* was used to calculate sample size [23]. A completely randomized treatment-control design was chosen. The total number of probe sets (genes) on the single microarray was 32323 (GeneChip Human Gene 1.0 ST Arrays – Affymetrix) and the number of genes that were not differentially expressed but falsely declared by the test procedure to be differentially expressed (number of false positives) was assumed to be about 1%. To simplify calculations, normal distribution of normalized gene expression values, the equality of variances and no interaction among genes were assumed. Based on preliminary data we anticipated that the variance of log-transformed gene expression levels should be equal to 0.6. The inclusion of 94 newborns (47 in each group) had power equal to 90% for detecting differentially expressed genes if significant gene expression difference was defined as fold expression change equal or greater than 1.5. 

Basic demographic data were compared using the one-way analysis of variance or Kruskal-Wallis analysis of variance as appropriate. Qualitative values were compared using the chi-square test. 

Neonatal data used for the study was recorded daily during hospitalization in NICU in a prospective manner and stored in computer databases. For the purpose of the study the following data was used: sex, birthweight, gestational age, intrauterine growth parameters, Apgar score, incidence of preeclampsia, maternal diabetes, preterm rupture of membranes, chorionamnionitis, delivery type, delivery room management, presence of respiratory distress syndrome (RDS), length of mechanical ventilation, surfactant administration, use of ibuprofen for patent ductus arteriosus (PDA), PDA ligation, early- and late-onset septic episodes, ureaplasma infection, prevelance of intraventricular haemorrhage (IVH), periventricular leukomalacia (PVL), weight gain during NICU stay and length of hospitalization. 

The microarray data were pre-processed using the R/Bioconductor package *aroma.affymetrix* [24,25]. For normalization we used Robust Multiarray Average (RMA) [26]. Quality control was performed by investigating Principal Component Analysis (PCA), *Relative Log Expression* (RLE) and Normalized Unscaled Standard Error (NUSE) plots. None of the arrays were discarded due to poor quality. Details of quality control analysis were presented in the online supplemental file – Figure S1-S7 in Appendix S1

Moderated t-tests [27] were performed to detect the probes that were differentially expressed between groups, using the *limma* package [28] in the R statistical software. Multiple testing correction, using the Benjamini-Hochberg procedure was applied to control the false discovery rate (FDR) [29]. If a probe set had an corrected for multiple comparison p-value less than 0.01, it was declared significantly differentially expressed. 

The groups differed with respect to birthweight, gestational age, delivery type, gender, Apgar score, delivery room intubation, surfactant therapy, prenatal steroids, use of ibuprofen for patent ductus arteriosus (PDA), PDA ligation, length of mechanical ventilation). The next step of the analysis was designed to examine whether the differences in gene expression between the groups were due to the differences between the groups with respect to these factors. A linear model was fitted to the expression data for each probe, and moderated *t*-tests and *F*-statistics were computed for each contrast. For each probe set that was found to be differentially expressed between the groups in the first part of the analysis, we tested whether this expression was explained by the group indicator and/or by gestational age and/or delivery type, gender, Apgar score, delivery room intubation, surfactant therapy, prenatal steroids, patent ductus arteriosus, length of mechanical ventilation using the function *classifyTestsF* from the limma package. 

For the four categories of BPD we used Spearman’s rho statistic [30] to test whether there was a monotonic trend in gene expression between the categories.

DAVID annotation tools were used to explore which predefined gene sets were significantly enriched in one group compared to another [31,32]. The KEGG (Kyoto Encyclopedia of Genes and Genomes; www.genome.jp/kegg) and Biocarta pathways (www.biocarta.com) were selected for analysis. Genes differentially expressed between BPD patients and controls with p-values adjusted for multiple testing below 0.01 and with fold change greater than 1.5 were used as input for pathway enrichment analysis in DAVID.

The mean fold change values representing differences in expression between the BPD group and the control group with regard to each of the 14 genes selected for validation were compared between the microarray and the TaqMan Gene Expression experiments. The Student’s *t*-test was used for evaluating the statistical significance of observed differences between gene expression.

## Results

### Characteristic of the studied group

One hundred twenty newborns were included in the study. The mean birth weight was 1029g (SD: 290), and the mean gestational age was 27.8 weeks (SD: 2.5). The majority of the pregnancies were terminated by abrupt deliveries. Only 50 (42%) of the mothers received antenatal steroids. Forty-nine (41%) were born by vaginal delivery. One hundred fourteen neonates (95%) required respiratory support. Seventy-three (61%) newborns received surfactant treatment. During the follow-up period, nine newborns died before the end of the study, therefore 111 children were included in the final analysis. 

### Bronchopulmonary dysplasia

During the follow-up period, BPD was diagnosed in 68 (61%) infants, including 40 (36%) children with mild disease, 13 (12%) with moderate and 15 (13%) with severe BPD. Forty-three newborns served as a control group (no BPD). The infants in the BPD group were of significantly lower birthweight and gestational age. As shown in Table 1 more infants in the BPD group received surfactant treatment and had persistent ductus arteriosus. 

**Table 1:** Comparison of selected demographic data in the bronchopulmonary dysplasia (BPD) groups and in the control children.

	noBPD (n=43)	Mild BPD (n=40)	Moderate BPD (n=13)	Severe BPD (n=15)	P
Birth weight, g (x±SD)	1245±187	960±244	887±300	736±208	<0.001^a^
Gestational age (weeks) (x±SD)	29.8 ±1.4	26.8±1.9	26.8±2.5	25.6±2.4	<0.001^a^
Female gender	24 (56%)	16 (40%)	5 (38%)	5 (33%)	0.32^b^
Vaginal delivery / Cesarean section	11/32	20/20	7/6	8/7	0.02^b^
Multiple pregnancy	12 (28%)	7 (18%)	2 (15%)	2 (13%	0.51^b^
Small-for-gestational-age infant	4 (9%)	5 (13%)	1 (8%)	5 (33%)	0.1^b^
Maternal hypertension	8 (19%)	7 (18%)	2 (15%)	1 (7%)	0.74^b^
Maternal diabetes	3 (7%)	2 (5%)	0	1 (7%)	0.79^b^
Maternal fever/infection prior delivery	5 (12%)	7 (18%)	3 (23%)	4 (27%)	0.53^b^
1^st^ minute Apgar Score (Me; 25-75^th^ percentile)	5 (4-6)	4 (2-6)	2 (1-5)	2 (1-4)	0.01^c^
5^th^ minute Apgar Score (Me; 25-75^th^ percentile)	7 (6-8)	6 (5-7)	4 (3-5)	6 (5-7)	0.001^c^
Delivery room intubation	15 (35%)	28 (70%)	10 (77%)	15 (100%)	0.001^b^
Surfactant therapy	15 (35%)	29 (73%)	9 (69%)	14 (93%)	0.003^b^
Initial a/A ratio	0.36±0.2	0.37±0.6	0.07±0.4	0.19±0.1	0.06^a^
Prenatal steroids	23 (54%)	14 (35%)	3 (23%)	6 (40%)	0.15^b^
Ureaplasma infection	3 (7%)	8 (20%)	3 (23%)	4 (27%)	0.28^b^
Culture-proven sepsis	16 (37%)	19 (48%)	6 (46%)	6 (40%)	0.47^b^
Pharmacological closure of patent ductus arteriosus	14 (33%)	21 (53%)	9 (69%)	11 (73%)	0.014^b^
Surgical closure of patent ductus arteriosus	1 (2%)	8 (20%)	5 (38%)	9 (60%)	<0.001^b^
Length of mechanical ventilation (Me; 25-75^th^ percentile)	2 (0-4)	11 (6-30)	42 (35-51)	70 (44-108)	<0.001^c^

^a^p-value for one-way analysis of variance

^b^p-value for chi-square test

^c^p-value for Kruskal-Wallis analysis of variance

### Whole genome expression

All data was collected and analyzed in the adherence to the Minimal Information About a Microarray Experiment guidelines. All primary microarray data was submitted to GEO public repository and are accessible through GEO Series accession number GSE32472 (http://www.ncbi.nlm.nih.gov/geo/query/acc.cgi?acc=GSE32472).

A summary of the number of differentially expressed genes between the control and BPD patients is presented in Table 2. Overall 2086 genes were differentially expressed on the 5^th^ DOL, only 324 on the 14^th^ DOL, and 3498 on the 28^th^ DOL. Generally, in each of the three time points during the first month of life more genes were under-expressed than over-expressed in the BPD group. The difference in expression measured as a fold change ranged between 1.0 and 1.5 in the majority of genes. 

**Table 2:** Summary of the number of differentially expressed genes between bronchopulmonary dysplasia and control patients.

Number of differentially expressed genes		Day 5	Day 14	Day 28	Expressed in both day 5 and 14	Expressed in both day 5 and day 28	Expressed in both day 14 and day 28	Expressed in all three measurements
Adjusted p value<0.01	All	2086	324	3498	209	1216	259	197 (Pathway analysis 4)
	Over-expressed	881	96	1394	30	431	58	28
	Under-expressed	1205	228	2104	179	785	201	169
Adjusted p value<0.01 and fold change>1.5	All	**266 (Pathway analysis 1**)	53	**302 (Pathway analysis 2**)	46	**180 (Pathway analysis 3**)	47	45
	Over-expressed	179	12	237	6	106	9	7
	Under-expressed	87	41	65	40	74	38	38
Adjusted p value<0.01 and fold change >2.0	All	40	1	34	1	24	1	1
	Over-expressed	40	1	33	1	24	1	1
	Under-expressed	0	0	1	0	0	0	0

The results of the multivariate analysis are presented in the Tables 3 and 4. Among 2086 genes differentially expressed on the 5th day of life, only 255 were differentially expressed due to BPD alone. Most of the genes were differentially expressed due to the interaction of two factors: BPD and gestational age. Similar results were obtained by the analysis of genes differentially expressed on the 14^th^ and 28^th^ day of life. The observed differences in gene expression were moderate, only in a few cases the fold change values were higher than 1.5. The heatmaps from clustering analysis of genes with known gene symbol were presented in the online data supplement - Figures S8-S10 in Appendix S1.

**Table 3:** Number of differentially expressed genes between the BPD and control group – results of multivariate analyses.

Model	Day 5 Adjusted p value <0.01	Day 14 Adjusted p value <0.01	Day 28 Adjusted p value <0.01
**Univariate anlysis - BPD**	2086	324	3498
**BPD and Gestational age (GA)**			
BPD only	**255 (pathway analysis 5**)	16	**199 (pathway analysis 6**
BPD and GA	1583	209	2584
**BPD and GA and Prenatal steroids (PS)**			
BPD only	**293**	18	**213**
BPD and GA	961	145	1874
BPD and PS	52	1	24
BPD and GA and PS	559	22	408
**BPD and GA and Gender**			
BPD only	**247**	16	**178**
BPD and GA	1229	156	1724
BPD and Gender	41	1	65
BPD and GA and Gender	235	28	558
**BPD and GA and delivery room intubation (DRI)**			
BPD only	**280**	17	**197**
BPD and GA	1204	178	1762
BPD and DRI	43	0	45
BPD and GA and DRI	264	9	477
**BPD and GA and surfactant administration (SA)**			
BPD only	**227**	12	**165**
BPD and GA	997	85	1632
BPD and SA	75	5	77
BPD and GA and SA	582	73	607
**BPD and GA and 5^th^ min. Apgar score (AS)**			
BPD only	**229**	14	**203**
BPD and GA	404	92	1690
BPD and AS	137	6	41
BPD and GA and AS	1032	104	728
**BPD and GA and Patent ductus arteriosus (PDA)**			
BPD only	**241**	15	**182**
BPD and GA	781	86	1239
BPD and PDA	99	3	157
BPD and GA and PDA	652	100	1149
**BPD and GA and mechanical ventilation (MV)**			
BPD only	**264**	18	**155**
BPD and GA	1156	36	444
BPD and MV	43	12	476
BPD and GA and MV	324	152	1528

**Table 4:** Number of differentially expressed genes between the BPD and control group – results of multivariate analyses.

Model	Day 5 Adjusted p value <0.01 and fold change >1.5	Day 28 Adjusted p value <0.01 and fold change >1.5
Univariate anlysis - BPD	266	302
**BPD and Gestational age (GA)**		
BPD only	6	2
BPD and GA	243*	286

* included in pathway analysis 7

Tables S1, S2 and S3 in Appendix S1 present the one hundred genes with the highest difference in expression on the 5^th^, 14^th^ and 28^th^ DOL, respectively. Tables S4, S5 and S6 in Appendix S1 present results of multivariate analysis - the genes that were differentially expressed on the 5^th^, 14^th^ and 28^th^ DOL due to BPD alone. Among the over-expressed genes, there were some known factors contributing to the development of BPD such as growth factors and their receptors. In the group of under-expressed genes, the majority were involved in the inflammatory response.

### Pathway enrichment analysis

Based on the results presented in Tables 2, 3 and 4, seven pathway enrichment analysis were performed. A summary of the analysis is presented in Table 5. The pathway up-regulated in the group of BPD patients is the cell cycle pathway. The activation of the cell cycle pathway does not seem to be related to the maturity of the infant. Detailed results of the cell cycle activation pathway are presented in Table S7 in Appendix S1.

**Table 5:** Summary of the seven pathway analysis for the differentially expressed genes between the BPD and control group.

Input to pathway analysis	Over-expressed		Under-expressed	
	Pathway name	FDR (%)	Pathway name	FDR (%)
Genes differentially expressed between BPD patients and controls on the 5th DOL, adjusted for multiple comparison p<0.01; fold change >1.5	**Cell cycle**	3.71	**T cell receptor signaling pathway**	<0.0001
			**Cell adhesion molecules (CAMs)**	0.001
			**Primary immunodeficiency**	0.002
			**Intestinal immune network for IgA production**	0.015
			**Hematopoietic cell lineage**	0.018
			Allograft rejection	1.674
			Autoimmune thyroid disease	4.501
Genes differentially expressed between BPD patients and controls on the 28th DOL. adjusted for multiple comparison p<0.01; fold change >1.5	**Cytokine-cytokine receptor interaction**	1.47	**T cell receptor signaling pathway**	0.014
	Fc gamma R-mediated phagocytosis	6.60	**Primary immunodeficiency**	0.026
			**Cell adhesion molecules (CAMs)**	0.046
			**Hematopoietic cell lineage**	0.066
			Ribosome	0.070
			**Intestinal immune network for IgA production**	0.101
			Allograft rejection	0.759
			Autoimmune thyroid disease	2.087
			Viral myocarditis	5.261
			Asthma	8.705
Genes differentially expressed between BPD patients and controls in two measurements: 5th and 28th DOL; p-values adjusted for multiple testing, p <0.01; fold change >1.5	**Cytokine-cytokine receptor interaction**	1.43	**T cell receptor signaling pathway**	0.001
			**Primary immunodeficiency**	0.006
			**Hematopoietic cell lineage**	0.010
			**Cell adhesion molecules (CAMs)**	0.084
			**Intestinal immune network for IgA production**	0.645
Genes differentially expressed between BPD patients and controls in all three measurements; p-values adjusted for multiple testing, p<0.01	No pathway		**T cell receptor signaling pathway**	<0.0001
			**Primary immunodeficiency**	0.0003
			**Hematopoietic cell lineage**	0.005
			**Cell adhesion molecules (CAMs)**	4.348
Genes differentially expressed between BPD patients and controls on the 5th DOL; p-values adjusted for multiple testing, p<0.01; no fold change restriction; included only genes differentially expressed due to BPD alone	**Cell cycle**	0.66	No pathway	
Genes differentially expressed between BPD patients and controls on the 28th DOL, p-values adjusted for multiple testing p<0.01; no fold change restriction; included only genes differentially expressed due to BPD alone	No pathway		No pathway	
Genes differentially expressed between BPD patients and controls on the 5th DOL; p-values adjusted for multiple testing p<0.01; fold change > 1.5; excluded genes differentially expressed not due to BPD (based on linear model including BPD. gestational age, gender and prenatal steroids as contributing factors for different gene expression)	**Cell cycle**	2.69	**T cell receptor signaling pathway**	<0.0001
			**Cell adhesion molecules (CAMs)**	0.0017
			**Primary immunodeficiency**	0.0027
			**Intestinal immune network for IgA production**	0.015
			**Hematopoietic cell lineage**	0.018

Pathways with FDR value less than 10% are shown.

Four pathways were continuously down-regulated on the 5^th^, 14^th^ and 28^th^ day of life in the group of BPD patients. However, expression of the genes assigned to these pathways depended on both factors: immaturity and disease. Detailed results of the T cell receptor signaling pathways are presented in Table S8 in Appendix S1.

### Genes expression and BPD severity

To detect the presence of a monotonic trend in gene expression between the BPD severity groups Spearman’s rho statistic was used. 1699 genes presented a monotone trend on the 5^th^, 1223 on the 14^th^ and 4766 on the 28^th^ day of life. Genes presenting a monotone trend in gene expression with p-values adjusted for multiple testing below 0.01 were used as input for pathway enrichment analysis in DAVID. Pathway enrichment analysis revealed similar results to those obtained in the two major groups (no BPD and BPD), see Table 6. 

**Table 6:** Pathway enrichment analysis of the genes presented a monotone trend in the gene expression between the BPD severity groups.

Genes differentially expressed between BPD patients and controls selected for pathway analysis	Over-expressed		Under-expressed	
	Pathway name	FDR (%)	Pathway name	FDR (%)
5th DOL, p-values adjusted for multiple comparison p<0.01;	**Cell cycle**	0.11	**Primary immunodeficiency**	0.00007
			**T cell receptor signaling pathway**	0.00023
			**Hematopoietic cell lineage**	0.677
14th DOL, p-values adjusted for multiple comparison p<0.01;	**Fc gamma R-mediated phagocytosis**	3.32	**T cell receptor signaling pathway**	0.00001
			**Primary immunodeficiency**	0.00010
			**Hematopoietic cell lineage**	0.46630
28th DOL, p-values adjusted for multiple comparison p<0.01;	**Fc gamma R-mediated phagocytosis**	0.0005	Aminoacyl-tRNA biosynthesis	0.001
	Chemokine signaling pathway	0.28	RNA degradation	0.009
	Fc epsilon RI signaling pathway	0.48	**Primary immunodeficiency**	0.025
	Neurotrophin signaling pathway	0.94	Purine metabolism	0.06
	Regulation of actin cytoskeleton	0.97	Pyrimidine metabolism	0.07
	GnRH signaling pathway	1.27	Spliceosome	0.16
	Focal adhesion	4.24	**Intestinal immune network for IgA production**	1.15
	Lysosome	5.02	**T cell receptor signaling pathway**	1.36
	Leukocyte transendothelial migration	5.55	Nucleotide excision repair	1.38
	Insulin signaling pathway	6.51	RNA polymerase	1.62
	MAPK signaling pathway	7.48	Asthma	8.26

Pathways with FDR value less than 10% are shown.

### Real-time PCR validation

**Figure 1:**
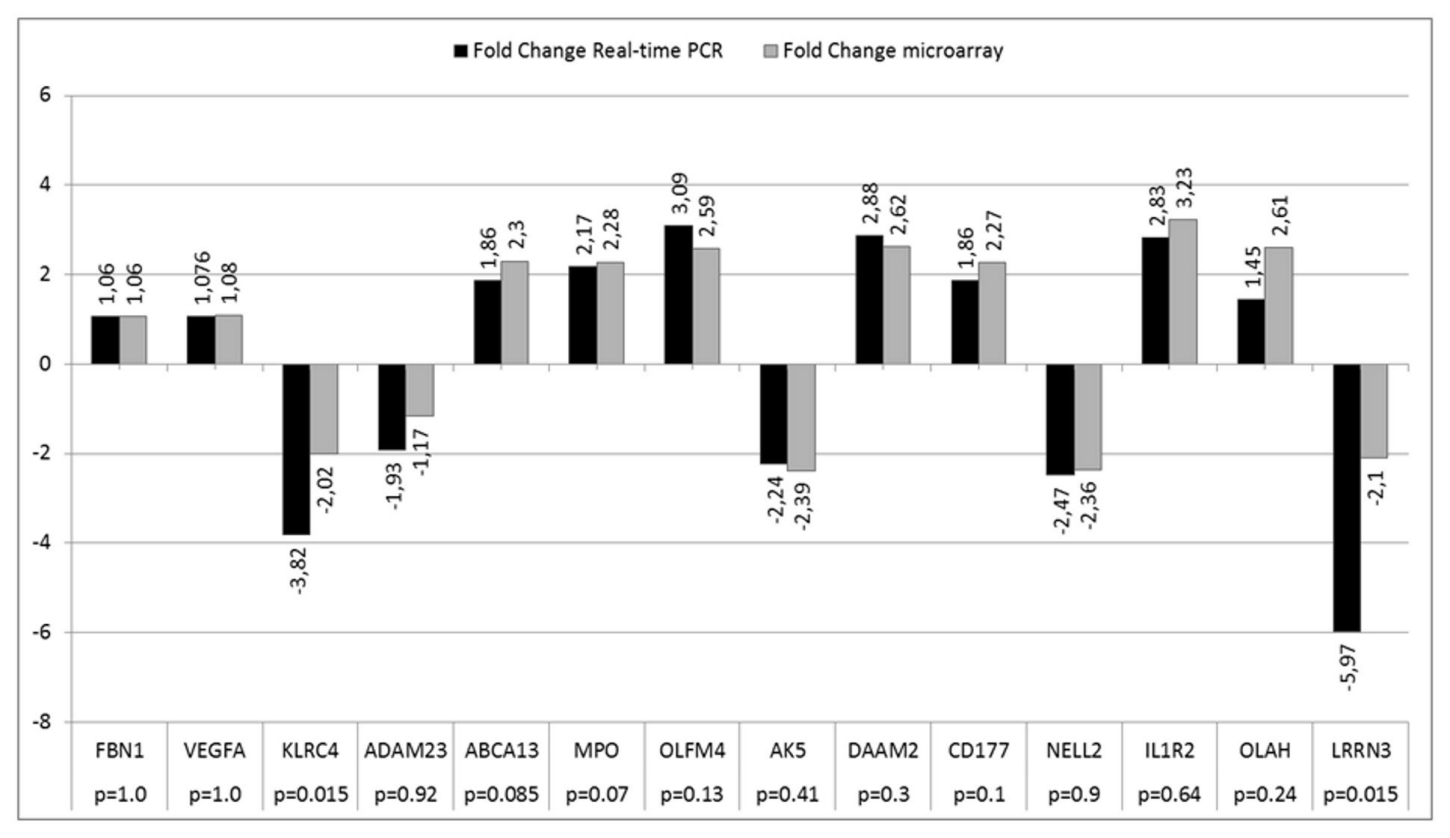
Validation of the microarray experiment by means of real time PCR. White bar – results of microarray evaluation, black bar – results of PCR validation. Table presents p value for *Student* *t-test*.

The microarray quality control was performed as recommended by Affymetrix. GeneChip Operating Software built-in algorithms were used for this purpose. All the microarrays fulfilled the quality criteria. The validation procedure did not reveal significant differences between the results obtained with use of microarrays compare to real time PCR technique Figure 1 gives details on the above analysis.

## Discussion

In this whole genome expression study of 5, 14 and 28 days old newborn children born before 32 weeks of gestation we have compared children who developed BPD to those without BPD. The nature of the study was mainly exploratory. We found a large number of differentially expressed genes between BPD and non BPD children. However, the difference in the expression measured as a fold change in the majority genes ranged between 1.0 and 1.5. The expression of only a few was twofold increased. Most of the genes were differentially expressed on the 5^th^ and 28^th^ day of life, and only a small number on the 14^th^ day of life. More genes in the BPD group, were under-expressed rather than over-expressed. When adjusting for gender, gestational age and prenatal steroid treatment, the largest group of differentially expressed genes, were differentially expressed due to the combination of two factors: gestational age and BPD. All this general data are in accordance with our understanding of BPD. Recently described attempts to create logistic regression models directed towards BPD prediction conducted on large patient cohorts point to prematurity as the most important risk factor of this condition [33,34].

Detailed analysis of the genes with the highest difference in expression was performed. Interestingly, in this group of genes we did not find many of the factors traditionally associated with the development of BPD (inflammatory mediators, growth factors).

A different method of evaluation of gene expression alterations is pathway enrichment analysis. This method can detect transcriptional programs that are distributed across an entire network of genes yet are subtle at the level of individual genes. Based on this method we found two groups of pathways which were differentially regulated at day 5, 14 and 28 after birth in infants with BPD. The first is the cell cycle pathway which seems to be a maturity independent pathway significantly up-regulated in children with BPD. The second group included pathways involved in inflammatory response. These pathways were down-regulated in children with BPD. The expression of genes involved in these pathways was related to both maturity and the severity of BPD.

### Cell cycle

Pathway enrichment analysis, based on gene expression fold change indicated the cell cycle pathway as one of the most up-regulated pathways among the BPD newborns. The genes assigned to this pathway with the highest fold change values are genes encoding the family of cyclins (*CYCA, CYCE* and *CYCB*) and cyclin related kinase 1 (*CDK1*). There was no difference in the expression of *CYCD* and *CDK4/CDK6*, as well as *CDK2* as a partner for cyclin E and A. This situation can be analyzed from several different aspects. The up-regulation of the cell cycle pathway might be considered an indicator of an increased cell mitosis rate and increased cell hyperplasia. 

In adult and newborn mice hyperoxia induced expression of p21 protein responsible for the inhibition of cyclin E dependent kinase activity. Moreover, results from some earlier animal experiments indicate that hyperoxia inhibits cell proliferation by two mechanisms; direct cell toxicity and modulation of G1/S phase transition [35].

The precise interpretation of our results is difficult in view of the highly specific character of the cell cycle and the still elusive knowledge on the gene interactions taking part in these complicated processes. The majority of cell cycle genes upregulated in the BPD newborns are involved in the regulation of G1/S transition (*CYCE*), S/G^2^ transition (*CYCA* and *CDK1*), and G2/M transition (*CYCB* and *CDK1*). The question can be raised, however, why neither *CYCD* nor *CDK4* and *CDK6* showed overexpression. S/G^2^ transition is under control of cyclin A and cyclin depended kinases CDK1 and CDK2. In our studied group overexpression of only *CYCA* and *CDK1* was observed. However, based on the experimental studies in CDK2 knockout mice [36] it was suggested that CDK2 is not required in the cell cycle progress and might be substituted by CDK1. Finally, the G2-M transition is controlled by the *CYCB/CDK1* complex whose increased expression was observed in the BPD group. 

As the level of cyclin B increases, it is bound to CDK1 and then transported to the nucleus promoting the beginning of the M phase. This process can be stopped by the phosphorylation of CDK. To protect CDK against this phosphorylation, a specific phosphatase cdc25 and Wee kinase are needed. In fact both of the above mentioned genes (*CDC25* and *WEE*) were found to be over-expressed in the BPD group. These results are therefore strongly indicative of increased mitotic activity of leukocytes originating from newborns who developed BPD. The *P53* gene representing the major “sensor” of DNA damage was under-expressed in the BPD group compared to the controls, and all genes coding for cyclin kinase inhibitory proteins like *P21*, *P27*, and *P57* showed comparable expression to those of the controls. This data might suggest that the group of BPD newborns was not exposed to, or was better protected against the influence of environmental factors potentially leading to DNA damage. 

However, it should be noted that the used microarray approach could reveal non-specific cell cycle pathway up-regulation. Some studies showed that there is a striking similarity between the gene expression responses to *a priori* very different phenomena. Abrupt temperature changes, oxidative stress or the addition of particular molecules are examples of such perturbations [37,38]. A change in the amount of nutrients is another example [39].

In summary, these data suggest strong cell mitotic activity in the group of children who developed BPD compared to the controls, with weaker indications of activation of DNA repair mechanisms.

### T cell receptor signaling pathway

T cells are activated upon the triggering of T cell receptors by antigen presenting cells. T cell activation induces a series of biochemical events that can differentially signal the naive T cell to enter into a pathway leading to the generation of effector T cells with the onset of rapid proliferation and production of effector cytokines; or enter into a state of antigenic non-responsiveness known as anergy or die by apoptosis [40]. The T cell response depends on the type of ligand bound to its’ receptors, time of co-operation, presence of co-receptors or co-inhibitors [41,42]. 

The production of cytokines is essentially controlled by transcription factors [43]. In our study, transcription factors and related pathways were under-expressed in children with BPD. When the differential expression analysis was adjusted for gender, gestational age and prenatal steroid treatment, it was revealed that not only immaturity is responsible for the down-regulation of genes in this pathway, but also disease severity. 

The significance of the finding decreased inflammatory genes expression might be unknown. However, we can speculate that decreased expression of T cell receptors may lead to relative anergy and can be a risk factor for bacterial translocation and further infection. These findings correspond with our knowledge that one of the risk factors of BPD is pulmonary infection [44]. Also, there are data indicating that expression of genes involved in the T cell receptors signaling pathway can be decreased by reactive oxygen species [45].

Analysis of single gene expression of the genes involved in these pathways revealed that some genes from this signaling pathway were over-expressed in BPD children. All the over-expressed genes in this signaling pathway belong to a classical part of the mitogen-activated protein kinase signaling pathway. These genes can be activated by growth factors or in response to pro-inflammatory stimuli. This signaling pathway is involved not only in the differentiation or proliferation of lymphocytes, but also of other cell lines.

### Limitations of the study

This type of study protocol raises the question of whether gene expression of white blood cells reflects gene expression in the lung. Investigators collaborating on the MuTHER project (Multiple Tissue Human Expression Resource) have addressed this issue by studying gene expression in different tissues in the same individuals [46]. The researchers collected skin, subcutaneous fat and peripheral blood from female twins. They found that overall 50 to 80% the loci identified in one tissue were estimated to have gene regulatory effects in a comparison tissue. Another interesting study was conducted by Rudkowska et al. [47]. The objective of this study was to validate the use of peripheral blood mononuclear cells for gene expression analysis as a marker of nutritional intervention as an alternative to skeletal muscle tissue biopsies. The author found that 88% (32,341) of transcripts were coexpressed in both tissues. Importantly, very strong correlation was observed between transcript expression levels of peripheral blood mononuclear cells and skeletal muscle tissue biopsies.

There is no data about the relationship between gene expression analysis in circulating cells and lung tissue, however we can only speculate, on the grounds that hypoxia/hyperoxia influences the organism of the newborn as a whole, that analysis of gene expression within the peripheral blood leukocytes which are characterized by high mitotic activity, may reflects gene expression in other cells. 

Of note is that the neonatal unit where the study was conducted accepts a highly selective group of newborns from primary and secondary referral centers. Thus, the study focused on a population with a high risk for BPD development. The specific character of the studied population might have influenced the results that were obtained; namely they were biased towards a statistically significant difference. The patient population may not be applicable to other NICUs (for example: the low rate of prenatal steroid use), and hence our results may not be generalizable. Another problem relates to the results of the statistical analysis in the case of multiple comparisons. If no correction was applied, multiple testing would increase the probability of a type I error to 1.0 (100%). Bearing this fact in mind, the correction for the multiple tests was performed, however at least 1% of false discovery rate can still be expected. The estimated power of the study to reveal a 1.5 fold change difference between the BPD and no BPD groups was 92.8%. 

It should be noted that the genome expression profiles detected in our study may be confounded by limitations related to the nature of biological systems which are dynamic and change constantly. The mRNAs are being synthesized and degraded at its own rate and the gene expression is measured during the experiment at a steady state. Thus, the presence of mRNA does not necessarily mean that it was just synthesized and the inability to detect certain transcripts may be due to their quick degradation. In addition, many biologically important changes do not manifest themselves in alterations of the RNA levels. Most of the cellular functions are performed by proteins and alteration to these functions can result not only from changes in protein levels but also from protein modifications [48].

## Conclusions

The results of this whole genome expression study revealed alteration of the expression of nearly 10% of the genome in BPD patients. Most of the differences in expression were associated with the degree of immaturity of the infant. However, based on pathway enrichment analysis we found pathways potentially involved in the pathogenesis of the disease. These results can be considered in two aspects. One, by reflecting our current knowledge and two, by providing some new information on the genes potentially involved in the BPD patomechanism. The results indicating on the overexpression of the pathways involving cytokine and theirs receptors, corroborates the generally accepted concept of the contribution of inflammatory response to the etiology of BPD. On other hand however, we observed lower expression of different immune response pathways, including T cell receptor pathways. These may point out on the relative immunedeficiency due to the extreme immaturity of the newborn. The second aspect of our results indicates some unknown facts, which may throw a new light on the BPD patomechanism. This refers to the significant overexpression of the cell cycle pathway in BPD patients. These findings should be considered with caution for two reasons. First, the over- and under-expression of the genes are dynamic process strictly dependent on the phase of the cell cycle, therefore the assessment performed at the single time point might be the resultant of different phases of the examined cells. Second, the final outcome represented by turning genes on or off is the result of concerted action of many other genes, mainly transcription factors, which activity might last too short to be determined. The above reservations do not change the fact, however, that the results are of sufficient interest to warrant further investigation on this subject.

## Supporting Information

Appendix S1 **Detailed description of microarray evaluation, tables S1-S8, figures S1-S10.**
(DOCX)
