# Supplementary material for: Gene Expression Profiling in Preterm Infants: New Aspects of Bronchopulmonary Dysplasia Development
Source: PLoS One. 2013 Oct 23;8(10):e78585. doi: 10.1371/journal.pone.0078585 (PMC3806835; doi:10.1371/journal.pone.0078585)

**Gene expression profiling in preterm infants: new aspects of bronchopulmonary dysplasia development**

Jacek J. Pietrzyk, Przemko Kwinta, Embjørg J. Wollen, Mirosław Bik – Multanowski, Anna Madetko – Talowska, Mateusz Jagła, Tomasz Tomasik, Ola D. Saugstad

Online Data Supplement

Methods

**Microarray analysis**

After obtaining written informed consent from the parents, blood samples (0.3 ml) were drawn from all the study participants on the 5th, 14th and 28th day of life (DOL) for the assessment of whole genome expression in peripheral blood leukocytes. Subsequently, Ficoll isopaque gradient centrifugation and RiboPure Blood Kit (Ambion, Life Technologies, Carlsbad, USA) were used for total RNA extraction. RNA concentration was measured with the use of NanoDrop spectrophotometer (NanoDrop ND-1000; Thermoscientific), and RNA quality was determined by 2100 Bioanalyzer (Agilent). RNA concentration ranged between 30,5-243,9 ng/µl (average 114,3 ng/µl).

100ng of total RNA was used for the microarray experiment. GeneChip Human Gene 1.0 ST Arrays (Affymetrix, Santa Clara, USA) were used. Whole microarray experiment was performed according to the manufacturers protocol. The Affymetrix GeneChip Whole Transcript (WT) sense Target Labeling Assay is designed to generate amplified and biotynylated sense-strand DNA targets from the entire expressed genome. Protocol is optimized for the use with the GeneChip Sense Target (ST) Arrays, where the probes are distributed throughout the entire lenght of each transcript.

At first 100ng of total RNA was mixed with Poly-A RNA controls (GeneChip Eukaryotic Poly-A Control Kit, Affymetrix, Santa Clara, USA). Subsequently cDNA sysnthesis was performed with random hexamers tagged with a T7 promoter sequence (GeneChip WT cDNA Synthesis and Amplification Kit, Sub-kit 1: GeneChip WT cDNA Synthesis Kit; Affymetrix, Santa Clara, USA). The double-stranded cDNA was then used as a template to produce many copies of antisense cRNA (GeneChip WT cDNA Synthesis and Amplification Kit, Sub-kit 2: GeneChip WT cDNA Amplification Kit; Affymetrix, Santa Clara, USA).

cRNA was treated with the cleanup procedure (GeneChip IVT cRNA, cDNA Cleanup Kit; Affymetrix, Santa Clara, USA). Subsequently cRNA yield was determined by spectrophotometric measurement (NanoDrop ND-1000; Thermoscientific).

10µg of cRNA was then used in the second cycle of cDNA synthesis. The random hexamers were used to prime riverse transcription of the cRNA to produce single-stranded DNA in the sense orientation (GeneChip WT cDNA Synthesis and Amplification Kit, Sub-kit 1: GeneChip WT cDNA Synthesis Kit; Affymetrix, Santa Clara, USA). During this step of the procedure, in order to reproducibly fragment ssDNA, dUTP was incorporated.

Subsequently, ssDNA was then proceed with the cleanup procedure (GeneChip IVT cRNA, cDNA Cleanup Kit; Affymetrix, Santa Clara, USA). DNA yield was determined by spectrophotometric measurement (NanoDrop ND-1000; Thermoscientific).

5,5µg of single-stranded DNA was treated with a combination of two enzymes: uracil DNA glycosidase (UDG) and apurinic/apyrimidinic endonuclease 1 (APE 1), specifically recognizing the dUTP nucleotides and breaking the DNA strand (GeneChip WT Terminal Labeling Kit; Affymetrix, Santa Clara, USA). Subsequently, fragmented DNA was labeled by terminal deoxynucleotidyl transferase (TdT) with the DNA Labeling Reagent, that was covalently linked to biotin (GeneChip WT Terminal Labeling Kit; Affymetrix, Santa Clara, USA).

Then fragmented and labeled DNA was hybridized with the GeneChip Human Gene 1.0 ST Array (Affymetrix, Santa Clara, USA). Apart from DNA, hybridization cocktail included: Eukaryotic Hybridization Controls (bioB, bioC, bioD, cre), Control Oligonucleotide B2 (GeneChip Hybridization Control Kit; Affymetrix, Santa Clara, USA), 2x Hybridization Mix, DMSO, water (GeneChip Hybridization, Wash and Stain Kit- Hybridization Module; Affymetrix, Santa Clara, USA). Hybridization was performed in 450C/ 60 rpm/ 17hours ±1hour at hybridization oven (GeneChip Hybridization Oven 640; Affymetrix, Santa Clara, USA).

After the hybridization, arrays were registered in GeneChip Operating Software (GCOS), and subsequently washed and stained with the use of GeneChip Fluidics Station 450 (Affymetrix, Santa Clara, USA) and FS450_0007 protocol (GeneChip Hybridization, Wash and Stain Kit-Stain Module; Wash Buffer A; Wash Buffer B; Affymetrix, Santa Clara, USA). After the wash protocol was finished arrays were scanned with the GeneChip Scanner 3000 7G (Affymetrix, Santa Clara, USA) controlled by GeneChip Operating Software.

For further statistical analysis **.cel files generated by GeneChip Operating Software were used.

**Quality control**

Quality control was performed by investigating Principal Component Analysis (PCA), Relative Log Expression (RLE) and Normalized Unscaled Standard Error (NUSE) plots.

*The Normalized Unscaled Standard Error (NUSE) is the individual probe error fitting the Probe-Level Model (the PLM models expression measures using a M-estimator robust regression). The NUSE values are standardized at the probe-set level across the arrays: median values for each probe-set are set to 1. The boxplots allow checking (1) if all distributions are centered near 1 – typically an array with a boxplot centered around 1.1 shows bad quality and (2) if one array has globally higher spread of NUSE distribution than others, which may also be a sign of low quality.

The figures S1, S2 and S3 represent results of NUSE analysis for 3 measurements (5, 14 and 28 day of life).

Figure S1. The Normalized Unscaled Standard Error values for individual microarrays on the 5^th^ day of life. Black dot represents median value, white box represents inter-quartile range, the dotted line – minimum-maximum range.


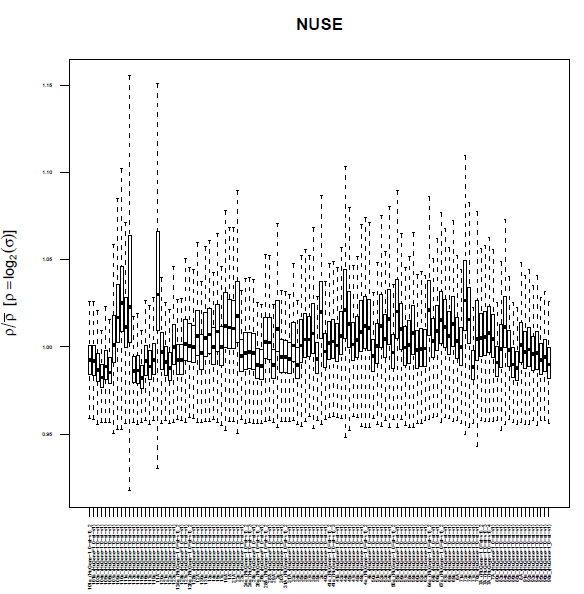


Figure S2. The Normalized Unscaled Standard Error values for individual microarrays on the 14^th^ day of life. Black dot represents median value, white box represents inter-quartile range, the dotted line – minimum-maximum range.

Figure S3. The Normalized Unscaled Standard Error values for individual microarrays on the 28th day of life. Black dot represents median value, white box represents inter-quartile range, the dotted line – minimum-maximum range.

*The Relative Log Expression (RLE) values are computed by calculating for each probe-set the ratio between the expression of a probe-set and the median expression of this probe-set across all arrays of the experiment. It is assumed that most probe-sets are not changed across the arrays, so it is expected that these ratios are around 0 on a log scale. The boxplots presenting the distribution of these log-ratios should then be centered near 0 and have similar spread. Other behavior would be a sign of low quality. The results of RLE analyses are presented on the Figures S4, S5 and S6

Figure S4. The Relative Log Expression (RLE) values for individual microarrays on the 5th day of life. Black dot represents median value, white box represents inter-quartile range, the dotted line – minimum-maximum range.

Figure S5. The Relative Log Expression (RLE) values for individual microarrays on the 14th day of life. Black dot represents median value, white box represents inter-quartile range, the dotted line – minimum-maximum range.


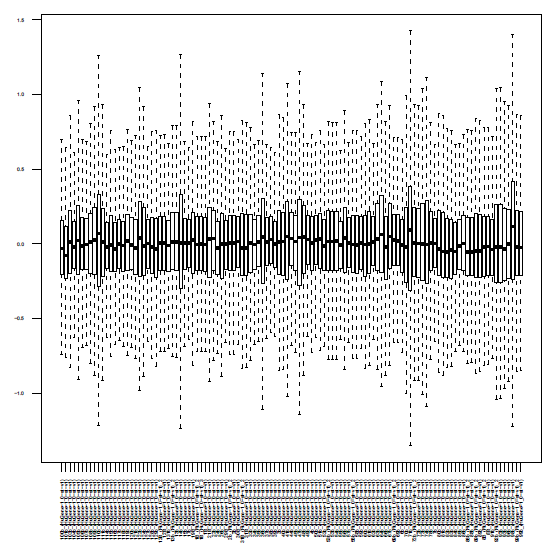


Figure S6. The Relative Log Expression (RLE) values for individual microarrays on the 28th day of life. Black dot represents median value, white box represents inter-quartile range, the dotted line – minimum-maximum range.

*The Principal Component Analysis shows the overall distance between the various arrays. If an array is very far from the other arrays from the same experimental group, it is a potential outlier (Figure S7).

Figure S7. The Principal Component Analysis. PC1 means principal component 1; PC2 means principal component 2; A (black circle) – measurements on the 5^th^ day of life; B – red triangle - measurements on the 14^th^ day of life; C – green cross - measurements on the 28^th^ day of life.

There were some potential outliers based on the above outlier measures (like number 19 in day group A (5DOL), and the third last in day group B (14DOL), but the signs where not unequivocal and we think that we here should be conservative about outlier removal as these are human data, and considerable individual variation is expected.

**Results**

Table S1. 100 genes with the highest difference in expression between BPD and control group on the 5 th day of life. Genes of interest marked with red and blue colors.

| gene assigment | gene abrevation | gene name | localization | | fold change | adjusted p value |
| --- | --- | --- | --- | --- | --- | --- |
| Up-regulated |  |  |  | |  |  |
| NM_006546 | IGF2BP1 | insulin-like growth factor 2 mRNA binding protein 1 | 17q21.32 | | 1.37 | 0.000015 |
| NM_000250 | MPO | myeloperoxidase | 17q23.1 | | 2.84 | 0.000015 |
| NM_003740 | KCNK5 | potassium channel, subfamily K, member 5 | 6p21 | | 1.47 | 0.000020 |
| NM_001004317 | LIN28B | lin-28 homolog B (C. elegans) | 6q21 | | 1.70 | 0.000021 |
| NM_004803 | SLC22A14 | solute carrier family 22, member 14 | 3p21.3 | | 1.19 | 0.000021 |
| NM_032536 | NTNG2 | netrin G2 | 9q34 | | 1.52 | 0.000021 |
| NM_022127 | SLC28A3 | solute carrier family 28 (sodium-coupled nucleoside transporter), member 3 | 9q22.2 | | 2.00 | 0.000021 |
| NM_000138 | FBN1 | fibrillin 1 | 15q21.1 | | 2.05 | 0.000021 |
| NM_144966 | FREM1 | FRAS1 related extracellular matrix 1 | 9p22.3 | | 1.32 | 0.000022 |
| NM_138799 | MBOAT2 | membrane bound O-acyltransferase domain containing 2 | 2p25.1 | | 1.61 | 0.000026 |
| NM_004633 | IL1R2 | interleukin 1 receptor, type II | 2q12 | | 3.20 | 0.000028 |
| NM_013230 | CD24 | CD24 molecule | 6q21 | | 2.44 | 0.000030 |
| NM_138399 | TMEM44 | transmembrane protein 44 | 3q29 | | 1.15 | 0.000031 |
| ENST00000290943 | ANKRD18B | ankyrin repeat domain 18B | 9p13.3 | | 1.39 | 0.000035 |
| NM_153615 | RGL4 | ral guanine nucleotide dissociation stimulator-like 4 | 22q11.23 | | 1.30 | 0.000037 |
| NM_138375 | CABLES1 | Cdk5 and Abl enzyme substrate 1 | 18q11.2 | | 1.35 | 0.000037 |
| NM_001001555 | GRB10 | growth factor receptor-bound protein 10 | 7p12.2 | | 1.61 | 0.000037 |
| NM_004776 | B4GALT5 | UDP-Gal:betaGlcNAc beta 1,4- galactosyltransferase, polypeptide 5 | 20q13.1-q13.2 | | 1.75 | 0.000037 |
| NM_002207 | ITGA9 | integrin, alpha 9 | 3p21.3 | | 1.89 | 0.000038 |
| NM_001924 | GADD45A | growth arrest and DNA-damage-inducible, alpha | 1p31.2 | | 1.52 | 0.000044 |
| Down-regulated |  |  |  |  | |  |
| NM_005450 | NOG | noggin | 17q22 | | 0.53 | 0.000000 |
| NM_174858 | AK5 | adenylate kinase 5 | 1p31 | | 0.56 | 0.000000 |
| NM_152785 | GCET2 | germinal center expressed transcript 2 | 3q13.2 | | 0.63 | 0.000000 |
| NR_024018 | ZNF30 | zinc finger protein 30 | 19q13.11 | | 0.72 | 0.000001 |
| NM_020379 | MAN1C1 | mannosidase, alpha, class 1C, member 1 | 1p35 | | 0.67 | 0.000003 |
| NM_018129 | PNPO | pyridoxamine 5'-phosphate oxidase | 17q21.32 | | 0.77 | 0.000006 |
| NM_145274 | TMEM99 | transmembrane protein 99 | 17q21.2 | | 0.74 | 0.000008 |
| NM_025228 | TRAF3IP3 | TRAF3 interacting protein 3 | 1q32 | | 0.74 | 0.000008 |
| NM_006994 | BTN3A3 | butyrophilin, subfamily 3, member A3 | 6p21.3 | | 0.61 | 0.000012 |
| AK296608 | C2orf89 | chromosome 2 open reading frame 89 | 2p11.2 | | 0.62 | 0.000014 |
| NM_006108 | SPON1 | spondin 1, extracellular matrix protein | 11p15.2 | | 0.75 | 0.000014 |
| NM_015946 | PELO | pelota homolog (Drosophila) | 5q11.2 | | 0.76 | 0.000014 |
| NM_003621 | PPFIBP2 | PTPRF interacting protein, binding protein 2 (liprin beta 2) | 11p15.4 | | 0.75 | 0.000015 |
| NM_006725 | CD6 | CD6 molecule | 11q13 | | 0.63 | 0.000016 |
| NM_001170553 | VSIG1 | V-set and immunoglobulin domain containing 1 | Xq22.3 | | 0.64 | 0.000017 |
| NM_019604 | CRTAM | cytotoxic and regulatory T cell molecule | 11q24.1 | | 0.60 | 0.000019 |
| BC005107 | C21orf105 | chromosome 21 open reading frame 105 | 21q22.3 | | 0.70 | 0.000019 |
| BC008360 | FAM113B | family with sequence similarity 113, member B | 12q13.11 | | 0.69 | 0.000020 |
| NM_004137 | KCNMB1 | potassium large conductance calcium-activated channel, subfamily M, beta member 1 | 5q34 | | 0.78 | 0.000020 |
| NM_002348 | LY9 | lymphocyte antigen 9 | 1q23.3 | | 0.66 | 0.000021 |
| NM_198083 | DHRS4L2 | dehydrogenase |  | | 0.76 | 0.000021 |
| NM_018961 | UBASH3A | ubiquitin associated and SH3 domain containing A | 21q22.3 | | 0.61 | 0.000021 |
| NM_001025199 | CHI3L2 | chitinase 3-like 2 | 1p13.3 | | 0.56 | 0.000021 |
| NM_000616 | CD4 | CD4 molecule | 12pter-p12 | | 0.63 | 0.000021 |
| NM_024600 | TMEM204 | transmembrane protein 204 | 16p13.3 | | 0.66 | 0.000021 |
| NM_015627 | LDLRAP1 | low density lipoprotein receptor adaptor protein 1 | 1p36-p35 | | 0.69 | 0.000021 |
| NM_020405 | PLXDC1 | plexin domain containing 1 | 17q21.1 | | 0.70 | 0.000021 |
| NM_001334 | CTSO | cathepsin O | 4q32.1 | | 0.71 | 0.000021 |
| NM_015041 | CLUAP1 | clusterin associated protein 1 | 16p13.3 | | 0.73 | 0.000021 |
| NM_024631 | C11orf61 | chromosome 11 open reading frame 61 | 11q24.2 | | 0.77 | 0.000021 |
| NM_001042533 | MINA | MYC induced nuclear antigen | 3q11.2 | | 0.78 | 0.000021 |
| NM_033411 | RWDD2A | RWD domain containing 2A | 6q14.2 | | 0.78 | 0.000021 |
| NM_014170 | GTPBP8 | GTP-binding protein 8 (putative) | 3q13.2 | | 0.78 | 0.000021 |
| NM_130759 | GIMAP1 | GTPase, IMAP family member 1 | 7q36.1 | | 0.68 | 0.000022 |
| NM_013441 | RCAN3 | RCAN family member 3 | 1p35.3-p33 | | 0.64 | 0.000022 |
| NM_000073 | CD3G | CD3g molecule, gamma (CD3-TCR complex) | 11q23 | | 0.53 | 0.000022 |
| NM_005164 | ABCD2 | ATP-binding cassette, sub-family D (ALD), member 2 | 12q11-q12 | | 0.57 | 0.000022 |
| NM_007047 | BTN3A2 | butyrophilin, subfamily 3, member A2 | 6p22.1 | | 0.60 | 0.000022 |
| NM_152272 | CHMP7 | CHMP family, member 7 | 8p21.3 | | 0.66 | 0.000022 |
| NM_014832 | TBC1D4 | TBC1 domain family, member 4 | 13q22.2 | | 0.75 | 0.000022 |
| NM_001099660 | LRRN3 | leucine rich repeat neuronal 3 | 7q31.1 | | 0.50 | 0.000023 |
| NM_024657 | MORC4 | MORC family CW-type zinc finger 4 | Xq22.3 | | 0.81 | 0.000024 |
| X58769 | TRAV8-3 | T cell receptor alpha variable 8-3 | 14q11 | | 0.57 | 0.000026 |
| NM_017933 | PID1 | phosphotyrosine interaction domain containing 1 | 2q36.3 | | 0.68 | 0.000026 |
| NM_181690 | AKT3 | v-akt murine thymoma viral oncogene homolog 3 (protein kinase B, gamma) | 1q44 | | 0.69 | 0.000026 |
| NM_005044 | PRKX | protein kinase, X-linked | Xp22.3 | | 0.80 | 0.000026 |
| NM_012092 | ICOS | inducible T-cell co-stimulator | 2q33 | | 0.56 | 0.000028 |
| NM_006139 | CD28 | CD28 molecule | 2q33 | | 0.58 | 0.000029 |
| NM_000155 | GALT | galactose-1-phosphate uridylyltransferase | 9p13 | | 0.74 | 0.000029 |
| NM_021004 | DHRS4 | dehydrogenase |  | | 0.76 | 0.000029 |
| NM_018090 | NECAP2 | NECAP endocytosis associated 2 | 1p36.13 | | 0.82 | 0.000029 |
| NM_003324 | TULP3 | tubby like protein 3 | 12p13.3 | | 0.83 | 0.000029 |
| NM_017918 | CCDC109B | coiled-coil domain containing 109B | 4q25 | | 0.64 | 0.000029 |
| NM_000074 | CD40LG | CD40 ligand | Xq26 | | 0.65 | 0.000031 |
| NM_004811 | LPXN | leupaxin | 11q12.1 | | 0.71 | 0.000031 |
| NM_018556 | SIRPG | signal-regulatory protein gamma | 20p13 | | 0.65 | 0.000033 |
| NM_002351 | SH2D1A | SH2 domain containing 1A | Xq25 | | 0.56 | 0.000033 |
| NM_013309 | SLC30A4 | solute carrier family 30 (zinc transporter), member 4 | 15q21.1\|15q21.1 | | 0.77 | 0.000033 |
| NM_022336 | EDAR | ectodysplasin A receptor | 2q13 | | 0.77 | 0.000034 |
| NM_007210 | GALNT6 | UDP-N-acetyl-alpha-D-galactosamine:polypeptide N-acetylgalactosaminyltransferase 6 (GalNAc-T6) | 12q13 | | 0.78 | 0.000035 |
| NM_017831 | RNF125 | ring finger protein 125 | 18q12.1 | | 0.70 | 0.000035 |
| NM_001838 | CCR7 | chemokine (C-C motif) receptor 7 | 17q12-q21.2 | | 0.56 | 0.000036 |
| NM_000733 | CD3E | CD3e molecule, epsilon (CD3-TCR complex) | 11q23 | | 0.60 | 0.000036 |
| NM_003726 | SKAP1 | src kinase associated phosphoprotein 1 | 17q21.32 | | 0.61 | 0.000036 |
| NM_014207 | CD5 | CD5 molecule | 11q13 | | 0.60 | 0.000037 |
| NM_145647 | WDR67 | WD repeat domain 67 | 8q24.13 | | 0.74 | 0.000037 |
| NM_003202 | TCF7 | transcription factor 7 (T-cell specific, HMG-box) | 5q31.1 | | 0.60 | 0.000038 |
| NM_004585 | RARRES3 | retinoic acid receptor responder (tazarotene induced) 3 | 11q23 | | 0.61 | 0.000038 |
| NM_198196 | CD96 | CD96 molecule | 3q13.13-q13.2 | | 0.57 | 0.000040 |
| NM_052838 | SEPT1 | septin 1 | 16p11.1 | | 0.59 | 0.000041 |
| NM_001034850 | FAM134B | family with sequence similarity 134, member B | 5p15.1 | | 0.67 | 0.000041 |
| NR_028049 | MTERFD2 | MTERF domain containing 2 | 2q37.3 | | 0.76 | 0.000041 |
| NM_018425 | PI4K2A | phosphatidylinositol 4-kinase type 2 alpha | 10q24 | | 0.77 | 0.000041 |
| NM_020827 | KIAA1430 | KIAA1430 | 4q35.1 | | 0.72 | 0.000042 |
| NM_018373 | SYNJ2BP | synaptojanin 2 binding protein | 14q24.2 | | 0.80 | 0.000042 |
| NM_020440 | PTGFRN | prostaglandin F2 receptor negative regulator | 1p13.1 | | 0.81 | 0.000042 |
| NM_001767 | CD2 | CD2 molecule | 1p13.1 | | 0.60 | 0.000044 |
| NM_003430 | ZNF91 | zinc finger protein 91 | 19p12 | | 0.69 | 0.000044 |
| NM_181671 | PITPNC1 | phosphatidylinositol transfer protein, cytoplasmic 1 | 17q24.2 | | 0.76 | 0.000044 |
| NM_183075 | CYP2U1 | cytochrome P450, family 2, subfamily U, polypeptide 1 | 4q25 | | 0.82 | 0.000044 |

Table S2. 100 genes with the highest difference in expression between BPD and control group on the 14 th day of life. Genes of interest marked with red and blue colors.

| gene assigment | gene abrevation | gene name | localization | fold change | adjusted p value |
| --- | --- | --- | --- | --- | --- |
| **Up-regulated** |  |  |  |  |  |
| AK299337 | FAM65C | family with sequence similarity 65, member C | 20q13.13 | 1.463513066 | 8.64261E-05 |
| NM_021978 | ST14 | suppression of tumorigenicity 14 (colon carcinoma) | 11q24-q25 | 1.350373315 | 8.64261E-05 |
| NM_004961 | GABRE | gamma-aminobutyric acid (GABA) A receptor, epsilon | Xq28 | 1.995891395 | 0.000254608 |
| NR_029638 | MIR224 | microRNA 224 | Xq28 | 2.756405393 | 0.000437577 |
| NM_003246 | THBS1 | thrombospondin 1 | 15q15 | 1.84037759 | 0.000511032 |
| NR_003530 | MEG3 | maternally expressed 3 (non-protein coding) | 14q32 | 1.517820192 | 0.000511032 |
| NM_000578 | SLC11A1 | solute carrier family 11 (proton-coupled divalent metal ion transporters), member 1 | 2q35 | 1.537690745 | 0.000783643 |
| NM_006546 | IGF2BP1 | insulin-like growth factor 2 mRNA binding protein 1 | 17q21.32 | 1.301924421 | 0.001043395 |
| NM_000362 | TIMP3 | TIMP metallopeptidase inhibitor 3 | 22q12.1-q13.2\|22q12.3 | 1.654582761 | 0.001159474 |
| NM_001013660 | FRRS1 | ferric-chelate reductase 1 | 1p21.2 | 1.334104997 | 0.001165638 |
| NM_021246 | LY6G6D | lymphocyte antigen 6 complex, locus G6D | 6p21.3 | 1.33941508 | 0.001395805 |
| NM_001128922 | LRRC32 | leucine rich repeat containing 32 | 11q13.5-q14 | 1.329137138 | 0.001397722 |
| NM_005539 | INPP5A | inositol polyphosphate-5-phosphatase, 40kDa | 10q26.3 | 1.254118008 | 0.00145622 |
| NM_001543 | NDST1 | N-deacetylase |  | 1.344836364 | 0.001534097 |
| NM_203370 | C3orf54 | chromosome 3 open reading frame 54 | 3p21.31 | 1.238249945 | 0.001748803 |
| NM_000420 | KEL | Kell blood group, metallo-endopeptidase | 7q33 | 1.591391557 | 0.001782737 |
| NM_000602 | SERPINE1 | serpin peptidase inhibitor, clade E (nexin, plasminogen activator inhibitor type 1), member 1 | 7q21.3-q22 | 1.39327066 | 0.001782737 |
| NM_020683 | ADORA3 | adenosine A3 receptor | 1p13.2 | 1.336343048 | 0.001782737 |
| NM_003749 | IRS2 | insulin receptor substrate 2 | 13q34 | 1.256629276 | 0.002271664 |
| NM_021246 | LY6G6D | lymphocyte antigen 6 complex, locus G6D | 6p21.3 | 1.39207576 | 0.002413264 |
| NM_021246 | LY6G6D | lymphocyte antigen 6 complex, locus G6D | 6p21.3 | 1.39207576 | 0.002413264 |
| NM_030923 | TMEM163 | transmembrane protein 163 | 2q21.3 | 1.270829766 | 0.002413264 |
| **Down-regulted** |  |  |  |  |  |
| NM_012092 | ICOS | inducible T-cell co-stimulator | 2q33 | 0.597748889 | 8.64261E-05 |
| NM_016388 | TRAT1 | T cell receptor associated transmembrane adaptor 1 | 3q13 | 0.589059946 | 0.000246509 |
| NM_001767 | CD2 | CD2 molecule | 1p13.1 | 0.617229475 | 0.000254608 |
| NM_006139 | CD28 | CD28 molecule | 2q33 | 0.649361817 | 0.00037763 |
| NM_018961 | UBASH3A | ubiquitin associated and SH3 domain containing A | 21q22.3 | 0.631853312 | 0.00037763 |
| NM_005546 | ITK | IL2-inducible T-cell kinase | 5q31-q32 | 0.61408989 | 0.00037763 |
| NM_000073 | CD3G | CD3g molecule, gamma (CD3-TCR complex) | 11q23 | 0.577828735 | 0.00037763 |
| NM_001935 | DPP4 | dipeptidyl-peptidase 4 | 2q24.3 | 0.639130533 | 0.000437577 |
| NM_006725 | CD6 | CD6 molecule | 11q13 | 0.690546347 | 0.000478397 |
| NM_002351 | SH2D1A | SH2 domain containing 1A | Xq25 | 0.582829067 | 0.000478397 |
| X58769 | TRAV8-3 | T cell receptor alpha variable 8-3 | 14q11 | 0.560228222 | 0.000478397 |
| NM_145251 | STYX | serine tyrosine interacting protein |  | 0.803176427 | 0.000511032 |
| NM_015344 | LEPROTL1 | leptin receptor overlapping transcript-like 1 | 8p21 | 0.753639114 | 0.000511032 |
| NM_018556 | SIRPG | signal-regulatory protein gamma | 20p13 | 0.665396653 | 0.000511032 |
| AK296608 | C2orf89 | chromosome 2 open reading frame 89 | 2p11.2 | 0.645397226 | 0.000511032 |
| NM_005164 | ABCD2 | ATP-binding cassette, sub-family D (ALD), member 2 | 12q11-q12 | 0.564719579 | 0.000511032 |
| NM_001039780 | CCNI2 | cyclin I family, member 2 | 5q31.1 | 0.830058496 | 0.000561541 |
| NM_198196 | CD96 | CD96 molecule | 3q13.13-q13.2 | 0.617939681 | 0.000561541 |
| NM_001164685 | THEMIS | thymocyte selection associated | 6q22.33 | 0.572622144 | 0.000561541 |
| NM_000733 | CD3E | CD3e molecule, epsilon (CD3-TCR complex) | 11q23 | 0.636745761 | 0.000783643 |
| NM_001099660 | LRRN3 | leucine rich repeat neuronal 3 | 7q31.1 | 0.531559519 | 0.000783643 |
| NM_006994 | BTN3A3 | butyrophilin, subfamily 3, member A3 | 6p21.3 | 0.64356997 | 0.000787891 |
| NM_006515 | SETMAR | SET domain and mariner transposase fusion gene | 3p26.1 | 0.844562616 | 0.000973333 |
| NM_145647 | WDR67 | WD repeat domain 67 | 8q24.13 | 0.737012819 | 0.000973333 |
| NM_004867 | ITM2A | integral membrane protein 2A | Xq13.3-Xq21.2 | 0.644354509 | 0.000973333 |
| NM_013441 | RCAN3 | RCAN family member 3 | 1p35.3-p33 | 0.714769125 | 0.001017294 |
| NM_198053 | CD247 | CD247 molecule | 1q22-q23 | 0.669983773 | 0.001040292 |
| NM_005450 | NOG | noggin | 17q22 | 0.5846575 | 0.001044893 |
| NM_002035 | KDSR | 3-ketodihydrosphingosine reductase | 18q21.3 | 0.794905737 | 0.001141098 |
| NM_005814 | GPA33 | glycoprotein A33 (transmembrane) | 1q24.1 | 0.668634438 | 0.001159474 |
| NM_001242 | CD27 | CD27 molecule | 12p13 | 0.631979532 | 0.001159474 |
| AK301287 | TRAJ17 | T cell receptor alpha joining 17 | 14q11 | 0.677218563 | 0.001165638 |
| NM_016269 | LEF1 | lymphoid enhancer-binding factor 1 | 4q23-q25 | 0.631304171 | 0.00131724 |
| NM_153236 | GIMAP7 | GTPase, IMAP family member 7 | 7q36.1 | 0.675306005 | 0.001360268 |
| NM_001017373 | SAMD3 | sterile alpha motif domain containing 3 | 6q23.1 | 0.609852386 | 0.001377285 |
| NM_006108 | SPON1 | spondin 1, extracellular matrix protein | 11p15.2 | 0.792482995 | 0.001384726 |
| NM_001040153 | SLAIN1 | SLAIN motif family, member 1 | 13q22.3 | 0.77721437 | 0.001395805 |
| BC008360 | FAM113B | family with sequence similarity 113, member B | 12q13.11 | 0.754169375 | 0.001395805 |
| NM_005127 | CLEC2B | C-type lectin domain family 2, member B | 12p13-p12 | 0.689311767 | 0.001395805 |
| NM_001128596 | TC2N | tandem C2 domains, nuclear | 14q32.12 | 0.668285391 | 0.001395805 |
| NM_001170553 | VSIG1 | V-set and immunoglobulin domain containing 1 | Xq22.3 | 0.665017035 | 0.001395805 |
| NM_174858 | AK5 | adenylate kinase 5 | 1p31 | 0.607500787 | 0.001414989 |
| NM_001334 | CTSO | cathepsin O | 4q32.1 | 0.73818369 | 0.001443464 |
| NM_005739 | RASGRP1 | RAS guanyl releasing protein 1 (calcium and DAG-regulated) | 15q14 | 0.683773208 | 0.001443464 |
| NM_012214 | MGAT4A | mannosyl (alpha-1,3-)-glycoprotein beta-1,4-N-acetylglucosaminyltransferase, isozyme A | 2q12 | 0.728431783 | 0.001534097 |
| BC030533 | TRBC1 | T cell receptor beta constant 1 | 7q34 | 0.731885737 | 0.001673183 |
| NM_138806 | CD200R1 | CD200 receptor 1 | 3q13.2 | 0.705608249 | 0.001673183 |
| NM_024421 | DSC1 | desmocollin 1 | 18q12.2\|18q12.1 | 0.635668526 | 0.001782737 |
| NM_152484 | ZNF569 | zinc finger protein 569 | 19q13.12 | 0.81956168 | 0.001788063 |
| NM_002145 | HOXB2 | homeobox B2 | 17q21.32 | 0.800475745 | 0.001788063 |
| NR_002920 | SNORA8 | small nucleolar RNA, H |  | 0.778664197 | 0.00180403 |
| NM_033160 | ZNF658 | zinc finger protein 658 | 9p13.1 | 0.833584472 | 0.001913212 |
| NM_022336 | EDAR | ectodysplasin A receptor | 2q13 | 0.783117789 | 0.001913212 |
| NM_017415 | KLHL3 | kelch-like 3 (Drosophila) | 5q31 | 0.680079085 | 0.001972058 |
| NM_033160 | ZNF658 | zinc finger protein 658 | 9p13.1 | 0.835756528 | 0.002103109 |
| NM_152785 | GCET2 | germinal center expressed transcript 2 | 3q13.2 | 0.691419446 | 0.00211744 |
| NM_138576 | BCL11B | B-cell CLL |  | 0.712275566 | 0.002118547 |
| NM_001034850 | FAM134B | family with sequence similarity 134, member B | 5p15.1 | 0.716104902 | 0.002134275 |
| NM_198066 | GNPNAT1 | glucosamine-phosphate N-acetyltransferase 1 | 14q22.1 | 0.78596692 | 0.002134582 |
| NM_015041 | CLUAP1 | clusterin associated protein 1 | 16p13.3 | 0.78600943 | 0.002137043 |
| NM_020347 | LZTFL1 | leucine zipper transcription factor-like 1 | 3p21.3 | 0.779660687 | 0.002137043 |
| NM_003726 | SKAP1 | src kinase associated phosphoprotein 1 | 17q21.32 | 0.659528636 | 0.002137043 |
| NM_014820 | TOMM70A | translocase of outer mitochondrial membrane 70 homolog A (S. cerevisiae) | 3q12.2 | 0.832313707 | 0.002228586 |
| BC005107 | C21orf105 | chromosome 21 open reading frame 105 | 21q22.3 | 0.752922847 | 0.002228586 |
| NM_001002295 | GATA3 | GATA binding protein 3 | 10p15 | 0.724318648 | 0.002228586 |
| NM_024600 | TMEM204 | transmembrane protein 204 | 16p13.3 | 0.720895452 | 0.002228586 |
| NM_015888 | HOOK1 | hook homolog 1 (Drosophila) | 1p32.1 | 0.685568087 | 0.002228586 |
| NM_002185 | IL7R | interleukin 7 receptor | 5p13 | 0.636093389 | 0.002228586 |
| NM_000732 | CD3D | CD3d molecule, delta (CD3-TCR complex) | 11q23 | 0.646993483 | 0.002271664 |
| NM_001098815 | KIAA0748 | KIAA0748 | 12q13.2 | 0.658546954 | 0.002282661 |
| NM_004529 | MLLT3 | myeloid |  | 0.702142505 | 0.002375868 |
| NM_024657 | MORC4 | MORC family CW-type zinc finger 4 | Xq22.3 | 0.822381663 | 0.002406842 |
| NM_016651 | DACT1 | dapper, antagonist of beta-catenin, homolog 1 (Xenopus laevis) | 14q23.1 | 0.805905357 | 0.002406842 |
| NM_000436 | OXCT1 | 3-oxoacid CoA transferase 1 | 5p13.1 | 0.78302925 | 0.002406842 |
| NM_000074 | CD40LG | CD40 ligand | Xq26 | 0.651537464 | 0.002406842 |
| NM_015946 | PELO | pelota homolog (Drosophila) | 5q11.2 | 0.801967752 | 0.002413264 |
| NM_152261 | C12orf23 | chromosome 12 open reading frame 23 | 12q23.3 | 0.798218838 | 0.002439322 |
| NM_018115 | SDAD1 | SDA1 domain containing 1 | 4q21.1 | 0.787269745 | 0.002439322 |

Table S3. 100 genes with the highest difference in expression between BPD and control group on the 28 th day of life. Genes of interest marked with red and blue colors.

| gene assigment | gene abrevation | gene name | localization | fold change | adjusted p value |
| --- | --- | --- | --- | --- | --- |
| **Up-regulated** |  |  |  |  |  |
| NM_002863 | PYGL | phosphorylase, glycogen, liver | 14q21-q22 | 1.870594049 | 1.9259E-06 |
| NM_005451 | PDLIM7 | PDZ and LIM domain 7 (enigma) | 5q35.3 | 1.482324108 | 1.9259E-06 |
| NM_007365 | PADI2 | peptidyl arginine deiminase, type II | 1p36.13 | 1.999851414 | 4.52798E-06 |
| NM_006825 | CKAP4 | cytoskeleton-associated protein 4 | 12q23.3 | 1.516790146 | 4.52798E-06 |
| NM_130385 | MRVI1 | murine retrovirus integration site 1 homolog | 11p15 | 1.411939815 | 5.17696E-06 |
| NM_001099270 | ZBTB34 | zinc finger and BTB domain containing 34 | 9q33.3 | 1.34605067 | 5.43736E-06 |
| NM_003830 | SIGLEC5 | sialic acid binding Ig-like lectin 5 | 19q13.3 | 1.919561281 | 6.1263E-06 |
| NM_012198 | GCA | grancalcin, EF-hand calcium binding protein | 2q24.2 | 1.880261212 | 6.1263E-06 |
| NM_006931 | SLC2A3 | solute carrier family 2 (facilitated glucose transporter), member 3 | 12p13.3 | 1.605129212 | 6.20309E-06 |
| NM_006546 | IGF2BP1 | insulin-like growth factor 2 mRNA binding protein 1 | 17q21.32 | 1.433733011 | 6.20309E-06 |
| NM_001130978 | DYSF | dysferlin, limb girdle muscular dystrophy 2B (autosomal recessive) | 2p13.3 | 2.116140801 | 9.40037E-06 |
| NM_001995 | ACSL1 | acyl-CoA synthetase long-chain family member 1 | 4q35 | 2.127368823 | 1.07511E-05 |
| NM_002964 | S100A8 | S100 calcium binding protein A8 | 1q21 | 1.53906669 | 1.07511E-05 |
| NM_023018 | NADK | NAD kinase | 1p36.33 | 1.319211271 | 1.30111E-05 |
| NM_174918 | C19orf59 | chromosome 19 open reading frame 59 | 19p13.2 | 1.956513164 | 1.38006E-05 |
| NM_004177 | STX3 | syntaxin 3 | 11q12.1 | 1.67556074 | 1.63703E-05 |
| NM_017983 | WIPI1 | WD repeat domain, phosphoinositide interacting 1 | 17q24.2 | 1.538860902 | 1.63703E-05 |
| NR_024151 | HSPA7 | heat shock 70kDa protein 7 (HSP70B) | 1q23.3 | 1.481754436 | 1.63703E-05 |
| NM_001164721 | PTAFR | platelet-activating factor receptor |  | 1.258541709 | 1.63703E-05 |
| NM_020406 | CD177 | CD177 molecule | 19q13.2 | 4.292280233 | 1.65596E-05 |
| NM_170776 | GPR97 | G protein-coupled receptor 97 | 16q21 | 2.488129813 | 1.65596E-05 |
| NM_181791 | GPR141 | G protein-coupled receptor 141 | 7p14.1 | 2.039760438 | 1.65596E-05 |
| NM_000904 | NQO2 | NAD(P)H dehydrogenase, quinone 2 | 6pter-q12 | 1.785967288 | 1.65596E-05 |
| NM_022746 | MOSC1 | MOCO sulphurase C-terminal domain containing 1 | 1q41 | 1.669749462 | 1.65596E-05 |
| NM_032564 | DGAT2 | diacylglycerol O-acyltransferase 2 | 11q13.5 | 1.652422342 | 1.65596E-05 |
| NM_005253 | FOSL2 | FOS-like antigen 2 | 2p23.3 | 1.486621544 | 1.65596E-05 |
| NM_001040022 | SIRPA | signal-regulatory protein alpha | 20p13 | 1.484621153 | 1.65596E-05 |
| NM_006317 | BASP1 | brain abundant, membrane attached signal protein 1 | 5p15.1 | 1.72445156 | 1.7583E-05 |
| NM_004795 | KL | Klotho | 13q12 | 1.339742421 | 1.7583E-05 |
| NM_004475 | FLOT2 | flotillin 2 | 17q11-q12 | 1.480742776 | 1.8343E-05 |
| NM_033334 | NR6A1 | nuclear receptor subfamily 6, group A, member 1 | 9q33.3 | 1.430635279 | 1.8343E-05 |
| NM_004668 | MGAM | maltase-glucoamylase (alpha-glucosidase) | 7q34 | 3.123189817 | 1.84035E-05 |
| NM_004776 | B4GALT5 | UDP-Gal:betaGlcNAc beta 1,4- galactosyltransferase, polypeptide 5 | 20q13.1-q13.2 | 1.842185257 | 1.91802E-05 |
| NM_001145808 | ITGAM | integrin, alpha M (complement component 3 receptor 3 subunit) | 16p11.2 | 1.477914603 | 1.91802E-05 |
| NM_014957 | DENND3 | DENN |  | 1.626312964 | 1.92762E-05 |
| NM_022468 | MMP25 | matrix metallopeptidase 25 | 16p13.3 | 2.008841807 | 2.03417E-05 |
| NM_031916 | ROPN1L | ropporin 1-like | 5p15.2 | 1.886012268 | 2.03954E-05 |
| NM_004664 | LIN7A | lin-7 homolog A (C. elegans) | 12q21 | 1.796897557 | 2.10359E-05 |
| NM_004130 | GYG1 | glycogenin 1 | 3q24-q25.1 | 1.847413218 | 2.12528E-05 |
| NM_004994 | MMP9 | matrix metallopeptidase 9 (gelatinase B, 92kDa gelatinase, 92kDa type IV collagenase) | 20q11.2-q13.1 | 3.041935093 | 2.14807E-05 |
| NM_203281 | BMX | BMX non-receptor tyrosine kinase | Xp22.2 | 2.200751213 | 2.14807E-05 |
| NM_001817 | CEACAM4 | carcinoembryonic antigen-related cell adhesion molecule 4 | 19q13.2 | 1.534258984 | 2.14807E-05 |
| NM_014326 | DAPK2 | death-associated protein kinase 2 | 15q22.31 | 1.439204129 | 2.14807E-05 |
| NM_199360 | TPD52L2 | tumor protein D52-like 2 | 20q13.2-q13.3 | 1.236255635 | 2.14807E-05 |
| NM_000395 | CSF2RB | colony stimulating factor 2 receptor, beta, low-affinity (granulocyte-macrophage) | 22q13.1 | 1.669309908 | 2.14946E-05 |
| NM_006254 | PRKCD | protein kinase C, delta | 3p21.31 | 1.390123147 | 2.14946E-05 |
| NM_022481 | ARAP3 | ArfGAP with RhoGAP domain, ankyrin repeat and PH domain 3 | 5q31.3 | 1.404622573 | 2.20928E-05 |
| NM_002000 | FCAR | Fc fragment of IgA, receptor for | 19q13.2-q13.4 | 1.764468641 | 2.30934E-05 |
| NM_144673 | CMTM2 | CKLF-like MARVEL transmembrane domain containing 2 | 16q21 | 1.655210223 | 2.60434E-05 |
| NM_001134338 | RNF24 | ring finger protein 24 | 20p13 | 1.527431661 | 2.60434E-05 |
| NM_001161531 | CSF2RA | colony stimulating factor 2 receptor, alpha, low-affinity (granulocyte-macrophage) | Xp22.32 and Yp11.3 | 1.444131445 | 2.60434E-05 |
| NM_002357 | MXD1 | MAX dimerization protein 1 | 2p13-p12 | 1.788363942 | 2.60833E-05 |
| NM_016546 | C1RL | complement component 1, r subcomponent-like | 12p13.31 | 1.47977404 | 2.72584E-05 |
| NM_153615 | RGL4 | ral guanine nucleotide dissociation stimulator-like 4 | 22q11.23 | 1.29739021 | 2.77282E-05 |
| NM_004665 | VNN2 | vanin 2 | 6q23-q24 | 1.88985539 | 2.80755E-05 |
| NM_006456 | ST6GALNAC2 | ST6 (alpha-N-acetyl-neuraminyl-2,3-beta-galactosyl-1,3)-N-acetylgalactosaminide alpha-2,6-sialyltransferase 2 | 17q25.1 | 1.653392055 | 2.80755E-05 |
| NM_024607 | PPP1R3B | protein phosphatase 1, regulatory (inhibitor) subunit 3B | 8p23.1 | 1.587307662 | 2.80755E-05 |
| NM_001099270 | ZBTB34 | zinc finger and BTB domain containing 34 | 9q33.3 | 1.362139909 | 2.80755E-05 |
| NM_181802 | UBE2C | ubiquitin-conjugating enzyme E2C | 20q13.12 | 1.333986151 | 2.80755E-05 |
| NM_000362 | TIMP3 | TIMP metallopeptidase inhibitor 3 | 22q12.1-q13.2\|22q12.3 | 1.774027908 | 2.99694E-05 |
| NM_005621 | S100A12 | S100 calcium binding protein A12 | 1q21 | 1.992767133 | 3.07319E-05 |
| NR_003530 | MEG3 | maternally expressed 3 (non-protein coding) | 14q32 | 1.556396889 | 3.08822E-05 |
| NM_001815 | CEACAM3 | carcinoembryonic antigen-related cell adhesion molecule 3 | 19q13.2 | 1.647327737 | 3.30549E-05 |
| NM_021958 | HLX | H2.0-like homeobox | 1q41 | 1.380582023 | 3.34167E-05 |
| NM_001193476 | SLC26A8 | solute carrier family 26, member 8 | 6p21 | 1.826846323 | 3.3637E-05 |
| NM_001462 | FPR2 | formyl peptide receptor 2 | 19q13.3-q13.4 | 1.985297669 | 3.43129E-05 |
| NM_000717 | CA4 | carbonic anhydrase IV | 17q23 | 1.458911982 | 3.43129E-05 |
| NM_004479 | FUT7 | fucosyltransferase 7 (alpha (1,3) fucosyltransferase) | 9q34.3 | 1.298742706 | 3.43129E-05 |
| NM_012335 | MYO1F | myosin IF | 19p13.3-p13.2 | 1.354469265 | 3.47127E-05 |
| Down-regulated |  |  |  |  |  |
| NM_174858 | AK5 | adenylate kinase 5 | 1p31 | 0.54696646 | 2.24756E-06 |
| NM_004655 | AXIN2 | axin 2 | 17q23-q24 | 0.744154478 | 6.1263E-06 |
| AK296608 | C2orf89 | chromosome 2 open reading frame 89 | 2p11.2 | 0.633838478 | 1.07511E-05 |
| NM_024512 | LRRC2 | leucine rich repeat containing 2 | 3p21.31 | 0.784491467 | 1.38006E-05 |
| NM_018961 | UBASH3A | ubiquitin associated and SH3 domain containing A | 21q22.3 | 0.607380199 | 1.38006E-05 |
| NR_024018 | ZNF30 | zinc finger protein 30 | 19q13.11 | 0.720781133 | 1.56816E-05 |
| NM_170740 | ALDH5A1 | aldehyde dehydrogenase 5 family, member A1 | 6p22 | 0.734703923 | 1.60816E-05 |
| NM_015147 | CEP68 | centrosomal protein 68kDa | 2p14 | 0.824088377 | 1.63703E-05 |
| NM_001039654 | ZNF550 | zinc finger protein 550 | 19q13.43 | 0.769331999 | 1.63703E-05 |
| NM_006725 | CD6 | CD6 molecule | 11q13 | 0.653916313 | 1.63703E-05 |
| NM_030915 | LBH | limb bud and heart development homolog (mouse) | 2p23.1 | 0.639457711 | 1.63703E-05 |
| NM_000074 | CD40LG | CD40 ligand | Xq26 | 0.634872953 | 1.63703E-05 |
| NM_020379 | MAN1C1 | mannosidase, alpha, class 1C, member 1 | 1p35 | 0.717702398 | 1.65596E-05 |
| NM_013441 | RCAN3 | RCAN family member 3 | 1p35.3-p33 | 0.649735953 | 1.65596E-05 |
| NM_152785 | GCET2 | germinal center expressed transcript 2 | 3q13.2 | 0.599182326 | 1.65596E-05 |
| NM_014872 | ZBTB5 | zinc finger and BTB domain containing 5 | 9p13.2 | 0.809269702 | 1.77575E-05 |
| NM_022917 | NOL6 | nucleolar protein family 6 (RNA-associated) | 9p13.3 | 0.796327921 | 1.8343E-05 |
| NM_005450 | NOG | noggin | 17q22 | 0.555027092 | 1.91802E-05 |
| NM_024662 | NAT10 | N-acetyltransferase 10 (GCN5-related) | 11p13 | 0.783100924 | 2.09445E-05 |
| NM_001098815 | KIAA0748 | KIAA0748 | 12q13.2 | 0.630648625 | 2.14807E-05 |
| NM_001025199 | CHI3L2 | chitinase 3-like 2 | 1p13.3 | 0.560868653 | 2.55434E-05 |
| NM_004753 | DHRS3 | dehydrogenase |  | 0.736298944 | 2.64433E-05 |
| NM_014976 | PDCD11 | programmed cell death 11 | 10q24.33 | 0.819310332 | 2.80755E-05 |
| NM_017875 | SLC25A38 | solute carrier family 25, member 38 | 3p22.1 | 0.768974135 | 2.80755E-05 |
| NM_006823 | PKIA | protein kinase (cAMP-dependent, catalytic) inhibitor alpha | 8q21.12 | 0.717092821 | 2.86379E-05 |
| NM_020347 | LZTFL1 | leucine zipper transcription factor-like 1 | 3p21.3 | 0.749028443 | 2.98737E-05 |
| NM_001979 | EPHX2 | epoxide hydrolase 2, cytoplasmic | 8p21 | 0.695601226 | 3.06651E-05 |
| NM_000733 | CD3E | CD3e molecule, epsilon (CD3-TCR complex) | 11q23 | 0.643596946 | 3.32408E-05 |
| NM_024600 | TMEM204 | transmembrane protein 204 | 16p13.3 | 0.695609002 | 3.39409E-05 |
| NM_183075 | CYP2U1 | cytochrome P450, family 2, subfamily U, polypeptide 1 | 4q25 | 0.789395545 | 3.43129E-05 |

Table S4. Differentially expressed genes between BPD and control group on the 5^th^ day of life – results of multivariate analysis

| Affymetrix ID | Gene symbol | Gene name | Fold change | P value | Corrected p value |
| --- | --- | --- | --- | --- | --- |
| 7947674 | RPS10 | ribosomal protein S10; ribosomal protein S10 pseudogene 4; ribosomal protein S10 pseudogene 11; ribosomal protein S10 pseudogene 22; ribosomal protein S10 pseudogene 7; ribosomal protein S10 pseudogene 13 | 0.66 | 0.00005 | 0.002 |
| 8086344 | CX3CR1 | chemokine (C-X3-C motif) receptor 1 | 0.67 | 0.00005 | 0.002 |
| 8139482 | SNORA5A | small nucleolar RNA, H/ACA box 5C; small nucleolar RNA, H/ACA box 5A; small nucleolar RNA, H/ACA box 5B | 0.72 | 0.00014 | 0.004 |
| 8003667 | SERPINF1 | serpin peptidase inhibitor, clade F (alpha-2 antiplasmin, pigment epithelium derived factor), member 1 | 0.72 | 0.00000 | 0.000 |
| 7893514 | NA |  | 0.73 | 0.00058 | 0.009 |
| 8038395 | NOSIP | nitric oxide synthase interacting protein | 0.75 | 0.00013 | 0.003 |
| 8116734 | LY86 | lymphocyte antigen 86 | 0.75 | 0.00016 | 0.004 |
| 7901052 | SNORD38B | small nucleolar RNA, C/D box 38A; small nucleolar RNA, C/D box 38B | 0.75 | 0.00023 | 0.005 |
| 7896700 | NA | CD86 molecule | 0.75 | 0.00008 | 0.002 |
| 7894968 | NA | eukaryotic translation initiation factor 3, subunit D | 0.75 | 0.00037 | 0.007 |
| 8121510 | RPF2 | brix domain containing 1 pseudogene; brix domain containing 1 | 0.76 | 0.00003 | 0.001 |
| 8045499 | HNMT | histamine N-methyltransferase | 0.76 | 0.00004 | 0.002 |
| 8119223 | FLJ45825 | ENSG00000204110 | 0.76 | 0.00062 | 0.010 |
| 8082035 | CD86 | CD86 molecule | 0.77 | 0.00022 | 0.005 |
| 7894586 | NA |  | 0.77 | 0.00012 | 0.003 |
| 8180245 | NA | ENSG00000204110 | 0.77 | 0.00013 | 0.003 |
| 8110755 | SLC12A7 | solute carrier family 12 (potassium/chloride transporters), member 7 | 0.77 | 0.00053 | 0.009 |
| 7927186 | RASSF4 | Ras association (RalGDS/AF-6) domain family member 4 | 0.78 | 0.00001 | 0.001 |
| 8180348 | XCL1 | chemokine (C motif) ligand 1 | 0.78 | 0.00034 | 0.007 |
| 8158998 | SNORD36C | ribosomal protein L7a pseudogene 70; ribosomal protein L7a; ribosomal protein L7a pseudogene 30; ribosomal protein L7a pseudogene 66; ribosomal protein L7a pseudogene 27; ribosomal protein L7a pseudogene 11; ribosomal protein L7a pseudogene 62 | 0.78 | 0.00021 | 0.005 |
| 8018169 | CD300LB | CD300 molecule-like family member b | 0.79 | 0.00000 | 0.000 |
| 7893249 | NA | eukaryotic translation initiation factor 3, subunit D | 0.79 | 0.00031 | 0.006 |
| 8042107 | NA | eukaryotic translation initiation factor 3, subunit F; similar to hCG2040283 | 0.79 | 0.00036 | 0.007 |
| 8002020 | TPPP3 | tubulin polymerization-promoting protein family member 3 | 0.79 | 0.00000 | 0.000 |
| 8006214 | ADAP2 | ArfGAP with dual PH domains 2 | 0.79 | 0.00003 | 0.001 |
| 8095139 | SRD5A3 | steroid 5 alpha-reductase 3 | 0.79 | 0.00024 | 0.005 |
| 8163002 | KLF4 | Kruppel-like factor 4 (gut) | 0.79 | 0.00035 | 0.007 |
| 7979416 | TIMM9 | translocase of inner mitochondrial membrane 9 homolog (yeast) | 0.79 | 0.00014 | 0.004 |
| 8159977 | RLN2 | relaxin 2 | 0.79 | 0.00003 | 0.001 |
| 8145470 | DPYSL2 | dihydropyrimidinase-like 2 | 0.80 | 0.00008 | 0.002 |
| 8017210 | AP1S2 | adaptor-related protein complex 1, sigma 2 subunit pseudogene; adaptor-related protein complex 1, sigma 2 subunit | 0.80 | 0.00025 | 0.005 |
| 7892742 | NA | asparaginyl-tRNA synthetase | 0.80 | 0.00001 | 0.000 |
| 7906079 | RAB25 | RAB25, member RAS oncogene family | 0.80 | 0.00011 | 0.003 |
| 7951397 | CASP1 | caspase 1, apoptosis-related cysteine peptidase (interleukin 1, beta, convertase) | 0.80 | 0.00034 | 0.007 |
| 8056995 | TTC30B | tetratricopeptide repeat domain 30B | 0.81 | 0.00008 | 0.002 |
| 8165995 | HCCS | holocytochrome c synthase (cytochrome c heme-lyase) | 0.82 | 0.00009 | 0.003 |
| 7906185 | APOA1BP | apolipoprotein A-I binding protein | 0.82 | 0.00044 | 0.008 |
| 7981824 | CYFIP1 | cytoplasmic FMR1 interacting protein 1 | 0.82 | 0.00026 | 0.005 |
| 7972055 | KCTD12 | potassium channel tetramerisation domain containing 12 | 0.82 | 0.00000 | 0.000 |
| 7913918 | UBXN11 | UBX domain protein 11 | 0.82 | 0.00005 | 0.002 |
| 8003922 | MED11 | mediator complex subunit 11 | 0.82 | 0.00050 | 0.009 |
| 8036136 | TMEM149 | transmembrane protein 149 | 0.82 | 0.00031 | 0.006 |
| 8000413 | NSMCE1 | non-SMC element 1 homolog (S. cerevisiae) | 0.82 | 0.00019 | 0.004 |
| 8033809 | ZNF846 | zinc finger protein 846 | 0.83 | 0.00036 | 0.007 |
| 8034299 | ELOF1 | elongation factor 1 homolog (S. cerevisiae) | 0.83 | 0.00051 | 0.009 |
| 8124459 | ZNF322A | zinc finger protein 322A | 0.83 | 0.00047 | 0.008 |
| 8052834 | C2orf42 | chromosome 2 open reading frame 42 | 0.83 | 0.00001 | 0.001 |
| 8059672 | NA | similar to barrier-to-autointegration factor; barrier to autointegration factor 1 | 0.83 | 0.00038 | 0.007 |
| 7952069 | C11orf60 | chromosome 11 open reading frame 60 | 0.83 | 0.00024 | 0.005 |
| 8090591 | PLXND1 | plexin D1 | 0.83 | 0.00053 | 0.009 |
| 7991126 | WDR73 | WD repeat domain 73 | 0.83 | 0.00000 | 0.000 |
| 7893842 | NA |  | 0.84 | 0.00056 | 0.009 |
| 8066247 | LOC388796 | hypothetical LOC388796 | 0.84 | 0.00027 | 0.006 |
| 8141768 | RASA4 | RAS p21 protein activator 4B | 0.84 | 0.00015 | 0.004 |
| 7896533 | NA | calpain, small subunit 1 | 0.84 | 0.00060 | 0.010 |
| 8080416 | LOC440957 | similar to CG32736-PA | 0.84 | 0.00057 | 0.009 |
| 8029360 | ZNF223 | zinc finger protein 223 | 0.84 | 0.00041 | 0.007 |
| 8138602 | DFNA5 | deafness, autosomal dominant 5 | 0.84 | 0.00001 | 0.001 |
| 7963774 | ZNF385A | zinc finger protein 385A | 0.84 | 0.00014 | 0.004 |
| 7966448 | TMEM116 | transmembrane protein 116 | 0.84 | 0.00000 | 0.000 |
| 8042576 | NAGK | N-acetylglucosamine kinase | 0.85 | 0.00051 | 0.009 |
| 8070708 | LOC284837 | hypothetical LOC284837 | 0.85 | 0.00010 | 0.003 |
| 8121312 | C6orf203 | chromosome 6 open reading frame 203 | 0.85 | 0.00012 | 0.003 |
| 8066384 | GTSF1L | gametocyte specific factor 1-like | 0.85 | 0.00001 | 0.001 |
| 7995739 | GNAO1 | guanine nucleotide binding protein (G protein), alpha activating activity polypeptide O | 0.85 | 0.00009 | 0.003 |
| 7963631 | RARG | retinoic acid receptor, gamma | 0.85 | 0.00002 | 0.001 |
| 7926345 | RPP38 | ribonuclease P/MRP 38kDa subunit | 0.85 | 0.00015 | 0.004 |
| 8148955 | C8orf33 | chromosome 8 open reading frame 33 | 0.85 | 0.00015 | 0.004 |
| 8028991 | CYP2S1 | cytochrome P450, family 2, subfamily S, polypeptide 1 | 0.85 | 0.00012 | 0.003 |
| 7915277 | MYCL1 | v-myc myelocytomatosis viral oncogene homolog 1, lung carcinoma derived (avian) | 0.86 | 0.00038 | 0.007 |
| 8110685 | LOC25845 | hypothetical LOC25845 | 0.86 | 0.00013 | 0.003 |
| 8030899 | ZNF766 | zinc finger protein 766 | 0.86 | 0.00035 | 0.007 |
| 8114567 | PFDN1 | prefoldin subunit 1 | 0.86 | 0.00040 | 0.007 |
| 7933180 | ZNF239 | zinc finger protein 239 | 0.86 | 0.00007 | 0.002 |
| 8037123 | POU2F2 | POU class 2 homeobox 2 | 0.86 | 0.00017 | 0.004 |
| 8180414 | ST6GALNAC1 | ST6 (alpha-N-acetyl-neuraminyl-2,3-beta-galactosyl-1,3)-N-acetylgalactosaminide alpha-2,6-sialyltransferase 1 | 0.86 | 0.00043 | 0.008 |
| 8018352 | SLC25A19 | solute carrier family 25 (mitochondrial thiamine pyrophosphate carrier), member 19 | 0.86 | 0.00004 | 0.001 |
| 8018774 | ST6GALNAC1 | ST6 (alpha-N-acetyl-neuraminyl-2,3-beta-galactosyl-1,3)-N-acetylgalactosaminide alpha-2,6-sialyltransferase 1 | 0.86 | 0.00048 | 0.008 |
| 7973427 | THTPA | thiamine triphosphatase | 0.86 | 0.00042 | 0.008 |
| 8004464 | TNFSF12-TNFSF13 | TNFSF12-TNFSF13 readthrough transcript; tumor necrosis factor (ligand) superfamily, member 12; tumor necrosis factor (ligand) superfamily, member 13 | 0.86 | 0.00022 | 0.005 |
| 8029423 | ZNF233 | zinc finger protein 233 | 0.86 | 0.00009 | 0.003 |
| 7898483 | ARHGEF10L | Rho guanine nucleotide exchange factor (GEF) 10-like | 0.86 | 0.00016 | 0.004 |
| 8025132 | ZNF557 | zinc finger protein 557 | 0.87 | 0.00033 | 0.006 |
| 8075906 | SSTR3 | somatostatin receptor 3 | 0.87 | 0.00050 | 0.009 |
| 7955156 | CCDC65 | coiled-coil domain containing 65 | 0.87 | 0.00021 | 0.005 |
| 7908397 | RGS13 | regulator of G-protein signaling 13 | 0.87 | 0.00054 | 0.009 |
| 7993680 | C16orf62 | chromosome 16 open reading frame 62 | 0.87 | 0.00007 | 0.002 |
| 8080804 | KCTD6 | potassium channel tetramerisation domain containing 6 | 0.87 | 0.00005 | 0.002 |
| 7975851 | C14orf179 | chromosome 14 open reading frame 179 | 0.87 | 0.00007 | 0.002 |
| 7963646 | AAAS | achalasia, adrenocortical insufficiency, alacrimia (Allgrove, triple-A) | 0.87 | 0.00037 | 0.007 |
| 7948829 | ZBTB3 | zinc finger and BTB domain containing 3 | 0.87 | 0.00028 | 0.006 |
| 7978174 | CHMP4A | chromatin modifying protein 4A | 0.87 | 0.00014 | 0.004 |
| 7941039 | RPS6KA4 | ribosomal protein S6 kinase, 90kDa, polypeptide 4 | 0.87 | 0.00023 | 0.005 |
| 7957126 | KCNMB4 | potassium large conductance calcium-activated channel, subfamily M, beta member 4 | 0.87 | 0.00025 | 0.005 |
| 7928208 | SLC29A3 | solute carrier family 29 (nucleoside transporters), member 3; Sp9 transcription factor homolog (mouse) | 0.87 | 0.00016 | 0.004 |
| 7940679 | TTC9C | tetratricopeptide repeat domain 9C | 0.87 | 0.00038 | 0.007 |
| 8036291 | ZNF565 | zinc finger protein 565 | 0.88 | 0.00002 | 0.001 |
| 8017143 | PTRH2 | peptidyl-tRNA hydrolase 2 | 0.88 | 0.00032 | 0.006 |
| 8008825 | DHX40 | similar to DEAH (Asp-Glu-Ala-His) box polypeptide 40; DEAH (Asp-Glu-Ala-His) box polypeptide 40 | 0.88 | 0.00015 | 0.004 |
| 7948718 | EML3 | echinoderm microtubule associated protein like 3 | 0.88 | 0.00032 | 0.006 |
| 8085481 | CHCHD4 | coiled-coil-helix-coiled-coil-helix domain containing 4 | 0.88 | 0.00039 | 0.007 |
| 8110043 | BNIP1 | BCL2/adenovirus E1B 19kDa interacting protein 1 | 0.88 | 0.00016 | 0.004 |
| 8024898 | FEM1A | fem-1 homolog a (C. elegans); similar to fem-1 homolog a (C.elegans); similar to fem-1 homolog a | 0.88 | 0.00047 | 0.008 |
| 8108579 | TMCO6 | transmembrane and coiled-coil domains 6 | 0.88 | 0.00000 | 0.000 |
| 7990400 | ULK3 | unc-51-like kinase 3 (C. elegans) | 0.88 | 0.00036 | 0.007 |
| 8033795 | ZNF561 | zinc finger protein 561; zinc finger protein 812 | 0.88 | 0.00037 | 0.007 |
| 7911730 | PANK4 | pantothenate kinase 4 | 0.89 | 0.00038 | 0.007 |
| 7998002 | ZNF276 | zinc finger protein 276 | 0.89 | 0.00030 | 0.006 |
| 8096917 | NA | ENSG00000211390 | 0.89 | 0.00034 | 0.007 |
| 8117922 | PRR3 | proline rich 3 | 0.89 | 0.00008 | 0.002 |
| 8043512 | ZNF2 | zinc finger protein 2 | 0.89 | 0.00001 | 0.001 |
| 8002237 | SLC7A6OS | solute carrier family 7, member 6 opposite strand | 0.89 | 0.00001 | 0.001 |
| 7996241 | SETD6 | SET domain containing 6 | 0.89 | 0.00040 | 0.007 |
| 7941639 | BBS1 | Bardet-Biedl syndrome 1 | 0.90 | 0.00023 | 0.005 |
| 8173154 | NA | ENSG00000211108 | 0.90 | 0.00016 | 0.004 |
| 8031857 | ZNF135 | zinc finger protein 135 | 0.90 | 0.00029 | 0.006 |
| 8143457 | NA | ENSG00000208334 | 0.90 | 0.00012 | 0.003 |
| 7997396 | ATMIN | ATM interactor | 0.92 | 0.00056 | 0.009 |
| 8033075 | RANBP3 | RAN binding protein 3 | 0.92 | 0.00017 | 0.004 |
| 8064042 | ARFGAP1 | ADP-ribosylation factor GTPase activating protein 1 | 0.92 | 0.00052 | 0.009 |
| 8087513 | TRAIP | TRAF interacting protein | 1.10 | 0.00049 | 0.008 |
| 7976412 | C14orf48 | chromosome 14 open reading frame 48 | 1.10 | 0.00021 | 0.005 |
| 8178470 | POU5F1 | ENSG00000198694 | 1.11 | 0.00029 | 0.006 |
| 8151123 | PTTG3P | pituitary tumor-transforming 3 | 1.11 | 0.00047 | 0.008 |
| 8002667 | PMFBP1 | polyamine modulated factor 1 binding protein 1 | 1.11 | 0.00046 | 0.008 |
| 8074701 | SLC7A4 | solute carrier family 7 (cationic amino acid transporter, y+ system), member 4 | 1.11 | 0.00057 | 0.009 |
| 7986975 | NA | ENSG00000210032 | 1.11 | 0.00056 | 0.009 |
| 7940108 | OR6Q1 | olfactory receptor, family 6, subfamily Q, member 1 | 1.12 | 0.00050 | 0.009 |
| 8063394 | PTPN1 | protein tyrosine phosphatase, non-receptor type 1 | 1.12 | 0.00018 | 0.004 |
| 7911269 | OR2M4 | olfactory receptor, family 2, subfamily M, member 4 | 1.12 | 0.00036 | 0.007 |
| 8163533 | FLJ31713 | hypothetical protein FLJ31713 | 1.12 | 0.00055 | 0.009 |
| 8021712 | NA | RNA, 7SL, cytoplasmic 2; RNA, 7SL, cytoplasmic 1 | 1.12 | 0.00033 | 0.006 |
| 7983979 | NA |  | 1.12 | 0.00051 | 0.009 |
| 8043393 | THNSL2 | threonine synthase-like 2 (S. cerevisiae) | 1.12 | 0.00032 | 0.006 |
| 7914000 | NR0B2 | nuclear receptor subfamily 0, group B, member 2 | 1.13 | 0.00045 | 0.008 |
| 8160771 | KIF24 | kinesin family member 24 | 1.13 | 0.00033 | 0.007 |
| 8088772 | NA |  | 1.13 | 0.00018 | 0.004 |
| 8146794 | PREX2 | phosphatidylinositol-3,4,5-trisphosphate-dependent Rac exchange factor 2 | 1.13 | 0.00029 | 0.006 |
| 8088550 | PRICKLE2 | prickle homolog 2 (Drosophila) | 1.13 | 0.00054 | 0.009 |
| 8096030 | NA | small nucleolar RNA, H/ACA box 75 | 1.13 | 0.00029 | 0.006 |
| 7897960 | AADACL3 | arylacetamide deacetylase-like 3 | 1.13 | 0.00015 | 0.004 |
| 7944302 | PHLDB1 | pleckstrin homology-like domain, family B, member 1 | 1.13 | 0.00007 | 0.002 |
| 8065122 | NA | small nucleolar RNA, C/D box 3B-1; small nucleolar RNA, C/D box 3B-2; small nucleolar RNA, C/D box 3A; small nucleolar RNA, C/D box 3C; small nucleolar RNA, C/D box 3D | 1.13 | 0.00011 | 0.003 |
| 8062461 | LBP | lipopolysaccharide binding protein | 1.13 | 0.00015 | 0.004 |
| 8091627 | NA | RNA, 7SL, cytoplasmic 2; RNA, 7SL, cytoplasmic 1 | 1.14 | 0.00008 | 0.002 |
| 7982299 | NA | ENSG00000103832 | 1.14 | 0.00037 | 0.007 |
| 8122598 | NA | RNA, U6 small nuclear 2; RNA, U6 small nuclear 1 | 1.14 | 0.00016 | 0.004 |
| 8023596 | NA | hCG1659830 | 1.14 | 0.00039 | 0.007 |
| 7938076 | OR52W1 | olfactory receptor, family 52, subfamily W, member 1 | 1.14 | 0.00004 | 0.002 |
| 7943998 | NNMT | nicotinamide N-methyltransferase | 1.14 | 0.00006 | 0.002 |
| 7975410 | NA | RNA, 7SL, cytoplasmic 2; RNA, 7SL, cytoplasmic 1 | 1.14 | 0.00033 | 0.006 |
| 7936994 | NA | RNA, 5S ribosomal 9; RNA, 5S ribosomal 13; RNA, 5S ribosomal 12; RNA, 5S ribosomal 11; RNA, 5S ribosomal 10; RNA, 5S ribosomal 17; RNA, 5S ribosomal 16; RNA, 5S ribosomal 15; RNA, 5S ribosomal 14; RNA, 5S ribosomal 1; RNA, 5S ribosomal 2; RNA, 5S ribosoma | 1.14 | 0.00055 | 0.009 |
| 8175438 | NA | ENSG00000208538 | 1.14 | 0.00056 | 0.009 |
| 7942771 | FLJ38894 | hypothetical protein LOC646029 | 1.14 | 0.00027 | 0.006 |
| 8012004 | NA | similar to Chromosome 17 open reading frame 49 | 1.15 | 0.00007 | 0.002 |
| 7946082 | UBQLNL | ubiquilin-like | 1.15 | 0.00011 | 0.003 |
| 8091780 | B3GALNT1 | beta-1,3-N-acetylgalactosaminyltransferase 1 (globoside blood group) | 1.15 | 0.00015 | 0.004 |
| 8074153 | ACR | acrosin | 1.15 | 0.00046 | 0.008 |
| 8155418 | FAM95B1 | ankyrin repeat domain 20 family, member A2 pseudogene; ankyrin repeat domain 20B; ankyrin repeat domain 20 family, member A4 | 1.15 | 0.00010 | 0.003 |
| 7995334 | NA | hypothetical gene supported by AK129756 | 1.15 | 0.00007 | 0.002 |
| 8083164 | NA | RNA, 7SK small nuclear | 1.15 | 0.00022 | 0.005 |
| 8032926 | PTPRS | protein tyrosine phosphatase, receptor type, S | 1.15 | 0.00040 | 0.007 |
| 8016139 | KIF18B | kinesin family member 18B | 1.15 | 0.00006 | 0.002 |
| 8128843 | DDO | D-aspartate oxidase | 1.16 | 0.00015 | 0.004 |
| 8092081 | NA | RNA, U1F1 small nuclear; RNA, U1C2 small nuclear; RNA, U1G2 small nuclear; RNA, U1C1 small nuclear; RNA, U1G3 small nuclear; RNA, U1A3 small nuclear; RNA, U1G1 small nuclear; RNA, U1A small nuclear | 1.16 | 0.00015 | 0.004 |
| 8071819 | NA | ENSG00000184490 | 1.16 | 0.00009 | 0.003 |
| 8082574 | TRH | thyrotropin-releasing hormone | 1.16 | 0.00002 | 0.001 |
| 7982206 | GOLGA9P | similar to Golgin subfamily A member 8-like protein 2; Golgin subfamily A member 8-like protein 1; Golgin subfamily A member 8-like protein 3; golgi autoantigen, golgin subfamily a, 9 pseudogene; similar to golgi autoantigen, golgin subfamily a, 8E | 1.16 | 0.00036 | 0.007 |
| 8137271 | ABP1 | amiloride binding protein 1 (amine oxidase (copper-containing)) | 1.17 | 0.00015 | 0.004 |
| 7897964 | NA | ENSG00000209466 | 1.17 | 0.00031 | 0.006 |
| 8078448 | NA | RNA, 7SL, cytoplasmic 2; RNA, 7SL, cytoplasmic 1 | 1.17 | 0.00009 | 0.003 |
| 7967454 | NA |  | 1.17 | 0.00019 | 0.004 |
| 7922326 | NA | microRNA 214; similar to hCG2041313 | 1.17 | 0.00010 | 0.003 |
| 8163084 | NA | 5.8S ribosomal RNA | 1.17 | 0.00019 | 0.004 |
| 8128888 | NA | RNA, U6 small nuclear 2; RNA, U6 small nuclear 1 | 1.17 | 0.00017 | 0.004 |
| 8047187 | CCDC150 | coiled-coil domain containing 150 | 1.17 | 0.00001 | 0.001 |
| 8093539 | WHSC1 | Wolf-Hirschhorn syndrome candidate 1 | 1.18 | 0.00041 | 0.007 |
| 8073858 | GTSE1 | G-2 and S-phase expressed 1 | 1.18 | 0.00010 | 0.003 |
| 8099193 | C4orf50 | chromosome 4 open reading frame 50 | 1.18 | 0.00009 | 0.003 |
| 8078772 | SLC22A14 | solute carrier family 22, member 14 | 1.19 | 0.00000 | 0.000 |
| 8040231 | C2orf48 | chromosome 2 open reading frame 48 | 1.19 | 0.00000 | 0.000 |
| 8175664 | NA | RNA, 5S ribosomal 9; RNA, 5S ribosomal 13; RNA, 5S ribosomal 12; RNA, 5S ribosomal 11; RNA, 5S ribosomal 10; RNA, 5S ribosomal 17; RNA, 5S ribosomal 16; RNA, 5S ribosomal 15; RNA, 5S ribosomal 14; RNA, 5S ribosomal 1; RNA, 5S ribosomal 2; RNA, 5S ribosoma | 1.19 | 0.00002 | 0.001 |
| 7982230 | GOLGA9P | similar to Golgin subfamily A member 8-like protein 2; Golgin subfamily A member 8-like protein 1; Golgin subfamily A member 8-like protein 3; golgi autoantigen, golgin subfamily a, 9 pseudogene; similar to golgi autoantigen, golgin subfamily a, 8E | 1.21 | 0.00005 | 0.002 |
| 8042211 | B3GNT2 | UDP-GlcNAc:betaGal beta-1,3-N-acetylglucosaminyltransferase 1; UDP-GlcNAc:betaGal beta-1,3-N-acetylglucosaminyltransferase 2 | 1.21 | 0.00042 | 0.008 |
| 7987114 | GOLGA9P | similar to Golgin subfamily A member 8-like protein 2; Golgin subfamily A member 8-like protein 1; Golgin subfamily A member 8-like protein 3; golgi autoantigen, golgin subfamily a, 9 pseudogene; similar to golgi autoantigen, golgin subfamily a, 8E | 1.21 | 0.00009 | 0.003 |
| 8022310 | NA | chromosome 18 open reading frame 58 | 1.21 | 0.00001 | 0.000 |
| 8172266 | NA | microRNA 221 | 1.22 | 0.00004 | 0.001 |
| 8108376 | NA | ENSG00000208810 | 1.22 | 0.00057 | 0.009 |
| 7938348 | WEE1 | WEE1 homolog (S. pombe) | 1.22 | 0.00021 | 0.005 |
| 8096002 | NA | ENSG00000210954 | 1.22 | 0.00045 | 0.008 |
| 8042040 | EML6 | ENSG00000143940 | 1.22 | 0.00061 | 0.010 |
| 7894092 | NA |  | 1.22 | 0.00056 | 0.009 |
| 8012475 | MYH10 | myosin, heavy chain 10, non-muscle | 1.23 | 0.00002 | 0.001 |
| 8005765 | WSB1 | WD repeat and SOCS box-containing 1 | 1.23 | 0.00025 | 0.005 |
| 7926896 | CKS1B | CDC28 protein kinase regulatory subunit 1B | 1.23 | 0.00012 | 0.003 |
| 8132118 | AQP1 | aquaporin 1 (Colton blood group) | 1.24 | 0.00001 | 0.001 |
| 8023080 | LOXHD1 | lipoxygenase homology domains 1 | 1.25 | 0.00046 | 0.008 |
| 8022043 | NA |  | 1.25 | 0.00020 | 0.005 |
| 7921429 | OR6N2 | olfactory receptor, family 6, subfamily N, member 2 | 1.25 | 0.00045 | 0.008 |
| 7981775 | NA | hypothetical protein DKFZp547L112 | 1.25 | 0.00000 | 0.000 |
| 8078153 | NA | RNA, 7SL, cytoplasmic 2; RNA, 7SL, cytoplasmic 1 | 1.25 | 0.00057 | 0.009 |
| 8059838 | HJURP | Holliday junction recognition protein | 1.25 | 0.00004 | 0.001 |
| 8081067 | HTR1F | 5-hydroxytryptamine (serotonin) receptor 1F | 1.26 | 0.00011 | 0.003 |
| 8112327 | CKS1B | CDC28 protein kinase regulatory subunit 1B | 1.26 | 0.00008 | 0.002 |
| 7927294 | ANTXRL | anthrax toxin receptor-like | 1.26 | 0.00001 | 0.001 |
| 8173506 | ERCC6L | excision repair cross-complementing rodent repair deficiency, complementation group 6-like | 1.26 | 0.00055 | 0.009 |
| 8154100 | VLDLR | very low density lipoprotein receptor | 1.27 | 0.00000 | 0.000 |
| 7894641 | NA |  | 1.28 | 0.00029 | 0.006 |
| 8064844 | PCNA | proliferating cell nuclear antigen | 1.28 | 0.00048 | 0.008 |
| 8054580 | BUB1 | budding uninhibited by benzimidazoles 1 homolog (yeast) | 1.28 | 0.00047 | 0.008 |
| 8045860 | PKP4 | plakophilin 4 | 1.28 | 0.00016 | 0.004 |
| 8146357 | MCM4 | minichromosome maintenance complex component 4 | 1.29 | 0.00019 | 0.004 |
| 7986068 | BLM | Bloom syndrome, RecQ helicase-like | 1.29 | 0.00004 | 0.001 |
| 8102800 | SLC7A11 | solute carrier family 7, (cationic amino acid transporter, y+ system) member 11 | 1.29 | 0.00062 | 0.010 |
| 8129763 | FAM54A | family with sequence similarity 54, member A | 1.30 | 0.00058 | 0.009 |
| 8021187 | SKA1 | chromosome 18 open reading frame 24 | 1.31 | 0.00017 | 0.004 |
| 8052382 | FANCL | Fanconi anemia, complementation group L | 1.31 | 0.00029 | 0.006 |
| 8160168 | FREM1 | FRAS1 related extracellular matrix 1 | 1.32 | 0.00000 | 0.000 |
| 7945014 | CHEK1 | CHK1 checkpoint homolog (S. pombe) | 1.32 | 0.00055 | 0.009 |
| 8144036 | XRCC2 | X-ray repair complementing defective repair in Chinese hamster cells 2 | 1.32 | 0.00001 | 0.001 |
| 8022974 | NA | RNA, U6 small nuclear 2; RNA, U6 small nuclear 1 | 1.33 | 0.00003 | 0.001 |
| 8013671 | SPAG5 | sperm associated antigen 5 | 1.33 | 0.00004 | 0.001 |
| 7922351 | LOC646870 | ENSG00000203739 | 1.34 | 0.00003 | 0.001 |
| 7908459 | CFH | complement factor H | 1.34 | 0.00028 | 0.006 |
| 7938366 | WEE1 | RNA, 7SL, cytoplasmic 2; RNA, 7SL, cytoplasmic 1 | 1.35 | 0.00015 | 0.004 |
| 8104758 | C5orf23 | chromosome 5 open reading frame 23 | 1.35 | 0.00053 | 0.009 |
| 8006187 | ATAD5 | ATPase family, AAA domain containing 5 | 1.35 | 0.00035 | 0.007 |
| 8095736 | AREG | amphiregulin; amphiregulin B | 1.35 | 0.00001 | 0.001 |
| 7938364 | WEE1 | ENSG00000209444 | 1.36 | 0.00014 | 0.004 |
| 8095110 | KIT | similar to Mast/stem cell growth factor receptor precursor (SCFR) (Proto-oncogene tyrosine-protein kinase Kit) (c-kit) (CD117 antigen); v-kit Hardy-Zuckerman 4 feline sarcoma viral oncogene homolog | 1.37 | 0.00021 | 0.005 |
| 7923965 | NA | ENSG00000208196 | 1.37 | 0.00042 | 0.008 |
| 7976818 | SNORD114-6 | small nucleolar RNA, C/D box 114-14; small nucleolar RNA, C/D box 113-1; small nucleolar RNA, C/D box 114-15; small nucleolar RNA, C/D box 114-16; small nucleolar RNA, C/D box 114-17; small nucleolar RNA, C/D box 114-10; small nucleolar RNA, C/D box 114-1 | 1.37 | 0.00053 | 0.009 |
| 7923189 | KIF14 | kinesin family member 14 | 1.37 | 0.00057 | 0.009 |
| 7895293 | NA |  | 1.39 | 0.00029 | 0.006 |
| 7987163 | NA | ENSG00000206125 | 1.39 | 0.00025 | 0.005 |
| 7918533 | ADORA3 | adenosine A3 receptor | 1.39 | 0.00001 | 0.001 |
| 7982248 | NA | ENSG00000212249; ENSG00000201398; ENSG00000199713; ENSG00000212594; ENSG00000202537; ENSG00000201809; ENSG00000200191; ENSG00000200496; ENSG00000212144; ENSG00000200026; ENSG00000207432; ENSG00000202269; ENSG00000212145; ENSG00000212581; ENSG00000207430; | 1.39 | 0.00025 | 0.005 |
| 7894882 | NA |  | 1.39 | 0.00057 | 0.009 |
| 7906930 | NUF2 | NUF2, NDC80 kinetochore complex component, homolog (S. cerevisiae) | 1.41 | 0.00057 | 0.009 |
| 8155214 | MELK | maternal embryonic leucine zipper kinase | 1.41 | 0.00031 | 0.006 |
| 8044021 | IL1RL1 | interleukin 1 receptor-like 1 | 1.41 | 0.00062 | 0.010 |
| 7982597 | THBS1 | thrombospondin 1 | 1.41 | 0.00053 | 0.009 |
| 7916898 | DEPDC1 | DEP domain containing 1 | 1.43 | 0.00018 | 0.004 |
| 8138822 | NA |  | 1.43 | 0.00004 | 0.002 |
| 7895250 | NA |  | 1.45 | 0.00056 | 0.009 |
| 7982269 | NA | ENSG00000212249; ENSG00000201398; ENSG00000199713; ENSG00000212594; ENSG00000202537; ENSG00000201809; ENSG00000200191; ENSG00000200496; ENSG00000212144; ENSG00000200026; ENSG00000207432; ENSG00000202269; ENSG00000212145; ENSG00000212581; ENSG00000207430; | 1.45 | 0.00010 | 0.003 |
| 7987025 | NA | ENSG00000212249; ENSG00000201398; ENSG00000199713; ENSG00000212594; ENSG00000202537; ENSG00000201809; ENSG00000200191; ENSG00000200496; ENSG00000212144; ENSG00000200026; ENSG00000207432; ENSG00000202269; ENSG00000212145; ENSG00000212581; ENSG00000207430; | 1.45 | 0.00010 | 0.003 |
| 8120838 | TTK | TTK protein kinase | 1.45 | 0.00008 | 0.002 |
| 7923086 | ASPM | asp (abnormal spindle) homolog, microcephaly associated (Drosophila) | 1.45 | 0.00057 | 0.009 |
| 7982757 | CASC5 | cancer susceptibility candidate 5 | 1.46 | 0.00009 | 0.003 |
| 8014248 | SLFN13 | schlafen family member 13 | 1.53 | 0.00001 | 0.001 |
| 7974920 | SYNE2 | spectrin repeat containing, nuclear envelope 2 | 1.54 | 0.00049 | 0.008 |
| 8095744 | AREG | amphiregulin; amphiregulin B | 1.57 | 0.00001 | 0.001 |
| 7937020 | MKI67 | antigen identified by monoclonal antibody Ki-67 | 1.59 | 0.00008 | 0.002 |
| 8144569 | NA | RNA, U6 small nuclear 2; RNA, U6 small nuclear 1 | 1.66 | 0.00043 | 0.008 |

Table S5. Differentially expressed genes between BPD and control group on the 14^th^ day of life – results of multivariate analysis

| Affymetrix ID | Gene symbol | Gene name | Fold change | P value | Corrected p value |
| --- | --- | --- | --- | --- | --- |
| 7896705 | NA | spleen tyrosine kinase | 0.75 | 0.00003 | 0.004 |
| 7906085 | LMNA | lamin A/C | 0.83 | 0.00001 | 0.003 |
| 7949971 | CPT1A | carnitine palmitoyltransferase 1A (liver) | 0.86 | 0.00001 | 0.003 |
| 7985268 | FAH | fumarylacetoacetate hydrolase (fumarylacetoacetase) | 0.86 | 0.00006 | 0.007 |
| 7992828 | IL32 | interleukin 32 | 1.16 | 0.00008 | 0.009 |
| 8005132 | MEIS3P1 | Meis homeobox 3 pseudogene 1 | 1.16 | 0.00009 | 0.009 |
| 8015914 | HDAC5 | histone deacetylase 5 | 1.19 | 0.00002 | 0.004 |
| 8040522 | MFSD2B | hypothetical protein LOC388931 | 1.19 | 0.00009 | 0.010 |
| 8051583 | CYP1B1 | cytochrome P450, family 1, subfamily B, polypeptide 1 | 1.24 | 0.00003 | 0.005 |
| 8059674 | GPR55 | G protein-coupled receptor 55 | 1.28 | 0.00002 | 0.004 |
| 8133459 | CLIP2 | CAP-GLY domain containing linker protein 2 | 1.34 | 0.00001 | 0.003 |
| 8135069 | SERPINE1 | serpin peptidase inhibitor, clade E (nexin, plasminogen activator inhibitor type 1), member 1 | 1.35 | 0.00002 | 0.004 |
| 8136940 | FAM115C | family with sequence similarity 115, member C; family with sequence similarity 115, member D (pseudogene) | 1.39 | 0.00000 | 0.002 |
| 8147891 | PKHD1L1 | polycystic kidney and hepatic disease 1 (autosomal recessive)-like 1 | 1.56 | 0.00002 | 0.004 |
| 8165974 | CLCN4 | chloride channel 4 | 1.67 | 0.00006 | 0.008 |
| 8175531 | CDR1 | cerebellar degeneration-related protein 1, 34kDa | 1.74 | 0.00001 | 0.003 |

Table S6. Differentially expressed genes between BPD and control group on the 28^th^ day of life – results of multivariate analysis

| Affymetrix ID | Gene symbol | Gene name | Fold change | P value | Corrected p value |
| --- | --- | --- | --- | --- | --- |
| 7961026 | OVOS | ovostatin; ovostatin 2 | 0.69 | 0.00083 | 0.009 |
| 7953873 | OVOS | ovostatin; ovostatin 2 | 0.71 | 0.00084 | 0.009 |
| 7893501 | NA |  | 0.73 | 0.00097 | 0.009 |
| 7894068 | NA | guanylate kinase 1 | 0.73 | 0.00023 | 0.004 |
| 8018708 | UBE2O | ubiquitin-conjugating enzyme E2O | 0.74 | 0.00019 | 0.003 |
| 8005166 | UBB | ubiquitin B | 0.74 | 0.00008 | 0.002 |
| 7899534 | EPB41 | erythrocyte membrane protein band 4.1 (elliptocytosis 1, RH-linked) | 0.75 | 0.00018 | 0.003 |
| 7892914 | NA |  | 0.75 | 0.00082 | 0.008 |
| 8113938 | ACSL6 | acyl-CoA synthetase long-chain family member 6 | 0.75 | 0.00047 | 0.006 |
| 8045009 | GYPC | glycophorin C (Gerbich blood group) | 0.77 | 0.00103 | 0.010 |
| 7894194 | NA |  | 0.77 | 0.00092 | 0.009 |
| 7895535 | NA |  | 0.77 | 0.00051 | 0.006 |
| 8150978 | CA8 | carbonic anhydrase VIII | 0.78 | 0.00054 | 0.006 |
| 7893648 | NA |  | 0.78 | 0.00011 | 0.002 |
| 8035773 | ZNF506 | zinc finger protein 506 | 0.78 | 0.00003 | 0.001 |
| 7892569 | NA | chromosome 2 open reading frame 24 | 0.79 | 0.00061 | 0.007 |
| 8015445 | NT5C3L | 5'-nucleotidase, cytosolic III-like | 0.79 | 0.00025 | 0.004 |
| 7963471 | KRT73 | keratin 73 | 0.79 | 0.00024 | 0.004 |
| 7969933 | BIVM | RNA, Ro-associated Y5 | 0.80 | 0.00084 | 0.009 |
| 8138088 | C7orf70 | chromosome 7 open reading frame 70 | 0.80 | 0.00001 | 0.000 |
| 8139820 | ZNF680 | zinc finger protein 680 | 0.80 | 0.00042 | 0.005 |
| 7893398 | NA |  | 0.81 | 0.00102 | 0.010 |
| 8040362 | LOC729992 | similar to heat shock 70kD protein binding protein; suppression of tumorigenicity 13 (colon carcinoma) (Hsp70 interacting protein) | 0.81 | 0.00020 | 0.003 |
| 7927082 | HSD17B7P2 | hydroxysteroid (17-beta) dehydrogenase 7 pseudogene 2 | 0.81 | 0.00032 | 0.005 |
| 8171879 | NA | similar to RAN binding protein 1; RAN binding protein 1 | 0.81 | 0.00079 | 0.008 |
| 7970716 | LNX2 | ligand of numb-protein X 2 | 0.82 | 0.00037 | 0.005 |
| 8180029 | HLA-DQB2 | major histocompatibility complex, class II, DQ beta 2 | 0.82 | 0.00038 | 0.005 |
| 8016285 | ARL17A | ADP-ribosylation factor-like 17 pseudogene 1; ADP-ribosylation factor-like 17 | 0.82 | 0.00086 | 0.009 |
| 7997281 | TERF2IP | telomeric repeat binding factor 2, interacting protein | 0.82 | 0.00088 | 0.009 |
| 8066247 | LOC388796 | hypothetical LOC388796 | 0.82 | 0.00001 | 0.001 |
| 8016300 | ARL17A | ADP-ribosylation factor-like 17 pseudogene 1; ADP-ribosylation factor-like 17 | 0.83 | 0.00056 | 0.007 |
| 7896185 | NA | DEAD (Asp-Glu-Ala-Asp) box polypeptide 5 | 0.83 | 0.00084 | 0.009 |
| 8099524 | LDB2 | LIM domain binding 2 | 0.83 | 0.00009 | 0.002 |
| 7950423 | NA | ENSG00000210384 | 0.83 | 0.00019 | 0.003 |
| 7895853 | NA | PRP8 pre-mRNA processing factor 8 homolog (S. cerevisiae) | 0.84 | 0.00030 | 0.004 |
| 7969559 | NA | prothymosin, alpha pseudogene 5 | 0.84 | 0.00007 | 0.002 |
| 8002087 | RANBP10 | RAN binding protein 10 | 0.84 | 0.00004 | 0.001 |
| 8117170 | NA | RNA, 7SK small nuclear | 0.84 | 0.00066 | 0.007 |
| 7967863 | ZNF605 | zinc finger protein 605 | 0.84 | 0.00087 | 0.009 |
| 8003283 | KLHDC4 | kelch domain containing 4 | 0.85 | 0.00026 | 0.004 |
| 8080416 | LOC440957 | similar to CG32736-PA | 0.85 | 0.00044 | 0.006 |
| 7913665 | NA | RNA, 7SL, cytoplasmic 2; RNA, 7SL, cytoplasmic 1 | 0.85 | 0.00061 | 0.007 |
| 7910261 | C1orf69 | chromosome 1 open reading frame 69 | 0.85 | 0.00012 | 0.002 |
| 8163271 | NA | hypothetical LOC100271832; RNA, Ro-associated Y5 pseudogene 10; RNA, Ro-associated Y1; RNA, Ro-associated Y4 pseudogene 7; RNA, Ro-associated Y4 pseudogene 19; RNA, Ro-associated Y3; hypothetical LOC100132111; RNA, Ro-associated Y4 | 0.85 | 0.00095 | 0.009 |
| 8091118 | NA |  | 0.86 | 0.00056 | 0.007 |
| 7898211 | DDI2 | regulatory solute carrier protein, family 1, member 1 | 0.86 | 0.00033 | 0.005 |
| 8055279 | NA | ENSG00000208350 | 0.86 | 0.00007 | 0.002 |
| 8052826 | SNORA36C | small nucleolar RNA, H/ACA box 36A; small nucleolar RNA, H/ACA box 36B; small nucleolar RNA, H/ACA box 36C (retrotransposed) | 0.86 | 0.00078 | 0.008 |
| 7959751 | ZNF664 | zinc finger protein 664 | 0.86 | 0.00001 | 0.001 |
| 8059770 | TIGD1 | tigger transposable element derived 1 | 0.86 | 0.00025 | 0.004 |
| 8060379 | PSMF1 | proteasome (prosome, macropain) inhibitor subunit 1 (PI31) | 0.86 | 0.00011 | 0.002 |
| 7995525 | NKD1 | naked cuticle homolog 1 (Drosophila) | 0.87 | 0.00001 | 0.000 |
| 7900446 | ZNF642 | zinc finger protein 642 | 0.87 | 0.00004 | 0.001 |
| 7985402 | UBE2Q2P3 | hypothetical LOC100134869 | 0.87 | 0.00080 | 0.008 |
| 7985444 | UBE2Q2P1 | similar to ubiquitin-conjugating enzyme E2Q 2 | 0.87 | 0.00061 | 0.007 |
| 7965871 | NA | RNA, 7SL, cytoplasmic 2; RNA, 7SL, cytoplasmic 1 | 0.88 | 0.00031 | 0.004 |
| 7919384 | NA | ENSG00000208867 | 0.88 | 0.00046 | 0.006 |
| 7934228 | ASCC1 | activating signal cointegrator 1 complex subunit 1 | 0.88 | 0.00082 | 0.008 |
| 8026007 | ZNF791 | zinc finger protein 791 | 0.88 | 0.00074 | 0.008 |
| 8139782 | NA | ENSG00000210926 | 0.88 | 0.00000 | 0.000 |
| 7996516 | PLEKHG4 | pleckstrin homology domain containing, family G (with RhoGef domain) member 4 | 0.88 | 0.00051 | 0.006 |
| 8025958 | ZNF440 | zinc finger protein 440 | 0.88 | 0.00048 | 0.006 |
| 7936134 | OBFC1 | oligonucleotide/oligosaccharide-binding fold containing 1 | 0.88 | 0.00047 | 0.006 |
| 7940698 | TAF6L | TAF6-like RNA polymerase II, p300/CBP-associated factor (PCAF)-associated factor, 65kDa | 0.89 | 0.00027 | 0.004 |
| 7934320 | DNAJC9 | DnaJ (Hsp40) homolog, subfamily C, member 9 | 0.89 | 0.00086 | 0.009 |
| 8127999 | NA | ENSG00000220831 | 0.89 | 0.00031 | 0.004 |
| 8122125 | NA | RNA, 7SK small nuclear | 0.89 | 0.00010 | 0.002 |
| 8061483 | CTD-2514C3.1 | hypothetical LOC100134868 | 0.89 | 0.00072 | 0.008 |
| 8065134 | NA | RNA, 7SK small nuclear | 0.89 | 0.00040 | 0.005 |
| 7988212 | ELL3 | elongation factor RNA polymerase II-like 3 | 0.90 | 0.00057 | 0.007 |
| 7931683 | DIP2C | DIP2 disco-interacting protein 2 homolog C (Drosophila) | 0.90 | 0.00059 | 0.007 |
| 8106765 | NA | ENSG00000211116 | 0.90 | 0.00011 | 0.002 |
| 8100123 | NA |  | 0.90 | 0.00013 | 0.002 |
| 7939173 | DEPDC7 | DEP domain containing 7 | 0.90 | 0.00089 | 0.009 |
| 7955250 | PRPF40B | PRP40 pre-mRNA processing factor 40 homolog B (S. cerevisiae) | 0.90 | 0.00074 | 0.008 |
| 8064500 | NA | hypothetical LOC100271832; RNA, Ro-associated Y5 pseudogene 10; RNA, Ro-associated Y1; RNA, Ro-associated Y4 pseudogene 7; RNA, Ro-associated Y4 pseudogene 19; RNA, Ro-associated Y3; hypothetical LOC100132111; RNA, Ro-associated Y4 | 0.91 | 0.00049 | 0.006 |
| 7934099 | NA | ENSG00000210247 | 0.91 | 0.00033 | 0.005 |
| 8045155 | NA | ENSG00000208419 | 0.91 | 0.00054 | 0.006 |
| 8000229 | C16orf65 | chromosome 16 open reading frame 65 | 0.91 | 0.00029 | 0.004 |
| 8048887 | NA | RNA, U6 small nuclear 2; RNA, U6 small nuclear 1 | 0.91 | 0.00083 | 0.009 |
| 7982390 | NA | small nucleolar RNA, C/D box 77 | 0.91 | 0.00023 | 0.004 |
| 7897449 | SPSB1 | splA/ryanodine receptor domain and SOCS box containing 1 | 0.91 | 0.00096 | 0.009 |
| 8084126 | NA | RNA, U6 small nuclear 2; RNA, U6 small nuclear 1 | 0.91 | 0.00096 | 0.009 |
| 7954701 | C12orf72 | chromosome 12 open reading frame 72 | 0.92 | 0.00083 | 0.009 |
| 8168620 | NA | small nucleolar RNA, C/D box 45A; small nucleolar RNA, C/D box 45B; small nucleolar RNA, C/D box 45C | 0.92 | 0.00054 | 0.006 |
| 7907535 | NA | ENSG00000208302 | 0.93 | 0.00024 | 0.004 |
| 8072131 | NA | RNA, U6 small nuclear 2; RNA, U6 small nuclear 1 | 0.93 | 0.00031 | 0.005 |
| 7967226 | IL31 | interleukin 31 | 1.09 | 0.00087 | 0.009 |
| 8153937 | SCRT1 | scratch homolog 1, zinc finger protein (Drosophila) | 1.09 | 0.00090 | 0.009 |
| 7924340 | NA | small nucleolar RNA, C/D box 3B-1; small nucleolar RNA, C/D box 3B-2; small nucleolar RNA, C/D box 3A; small nucleolar RNA, C/D box 3C; small nucleolar RNA, C/D box 3D | 1.09 | 0.00098 | 0.010 |
| 8015179 | KRTAP1-5 | ENSG00000204888 | 1.09 | 0.00041 | 0.005 |
| 8019588 | KRTAP1-5 | ENSG00000204888 | 1.09 | 0.00041 | 0.005 |
| 8015337 | KRT15 | keratin 15 | 1.09 | 0.00099 | 0.010 |
| 7972601 | NALCN | sodium leak channel, non-selective | 1.09 | 0.00062 | 0.007 |
| 8007603 | NA | small nucleolar RNA, C/D box 3B-1; small nucleolar RNA, C/D box 3B-2; small nucleolar RNA, C/D box 3A; small nucleolar RNA, C/D box 3C; small nucleolar RNA, C/D box 3D | 1.09 | 0.00056 | 0.007 |
| 8176442 | TBL1Y | transducin (beta)-like 1Y-linked | 1.10 | 0.00034 | 0.005 |
| 7971369 | KCTD4 | potassium channel tetramerisation domain containing 4 | 1.10 | 0.00067 | 0.007 |
| 7957386 | ACSS3 | acyl-CoA synthetase short-chain family member 3 | 1.10 | 0.00099 | 0.010 |
| 7928218 | CDH23 | cadherin-like 23 | 1.10 | 0.00062 | 0.007 |
| 8011808 | GPR172B | G protein-coupled receptor 172B | 1.11 | 0.00085 | 0.009 |
| 7938652 | INSC | inscuteable homolog (Drosophila) | 1.11 | 0.00083 | 0.009 |
| 7945296 | GLB1L3 | galactosidase, beta 1-like 3 | 1.11 | 0.00100 | 0.010 |
| 8079107 | KBTBD5 | kelch repeat and BTB (POZ) domain containing 5 | 1.11 | 0.00036 | 0.005 |
| 8095072 | NA | similar to COMM domain containing 5 | 1.11 | 0.00090 | 0.009 |
| 7953603 | C1S | complement component 1, s subcomponent | 1.11 | 0.00012 | 0.002 |
| 8060765 | PRND | prion protein 2 (dublet) | 1.11 | 0.00094 | 0.009 |
| 7938076 | OR52W1 | olfactory receptor, family 52, subfamily W, member 1 | 1.11 | 0.00094 | 0.009 |
| 8015189 | KRTAP1-3 | ENSG00000204887 | 1.11 | 0.00023 | 0.004 |
| 8019585 | KRTAP1-3 | ENSG00000204887 | 1.11 | 0.00023 | 0.004 |
| 7930162 | C10orf26 | chromosome 10 open reading frame 26 | 1.11 | 0.00096 | 0.009 |
| 8038785 | VSIG10L | hypothetical protein LOC147645 | 1.11 | 0.00010 | 0.002 |
| 8163019 | ACTL7B | actin-like 7B | 1.12 | 0.00098 | 0.010 |
| 8180219 | FLJ16171 | FLJ16171 protein | 1.12 | 0.00081 | 0.008 |
| 7917470 | NA | ENSG00000211185 | 1.12 | 0.00088 | 0.009 |
| 8027728 | HPN | hepsin | 1.12 | 0.00101 | 0.010 |
| 8074701 | SLC7A4 | solute carrier family 7 (cationic amino acid transporter, y+ system), member 4 | 1.12 | 0.00092 | 0.009 |
| 7905548 | SPRR3 | small proline-rich protein 3 | 1.12 | 0.00015 | 0.003 |
| 7906015 | NA | RNA, U6 small nuclear 2; RNA, U6 small nuclear 1 | 1.12 | 0.00090 | 0.009 |
| 8097126 | NA | hypothetical LOC100192379 | 1.12 | 0.00025 | 0.004 |
| 8129067 | NA | RNA, 5S ribosomal 9; RNA, 5S ribosomal 13; RNA, 5S ribosomal 12; RNA, 5S ribosomal 11; RNA, 5S ribosomal 10; RNA, 5S ribosomal 17; RNA, 5S ribosomal 16; RNA, 5S ribosomal 15; RNA, 5S ribosomal 14; RNA, 5S ribosomal 1; RNA, 5S ribosomal 2; RNA, 5S ribosoma | 1.12 | 0.00005 | 0.001 |
| 7906163 | RHBG | Rh family, B glycoprotein (gene/pseudogene) | 1.12 | 0.00062 | 0.007 |
| 8010780 | TEX19 | testis expressed 19 | 1.12 | 0.00101 | 0.010 |
| 8155083 | CA9 | carbonic anhydrase IX | 1.12 | 0.00087 | 0.009 |
| 8123437 | KIF25 | kinesin family member 25 | 1.12 | 0.00072 | 0.008 |
| 7963366 | KRT85 | keratin 85 | 1.13 | 0.00051 | 0.006 |
| 8044067 | SLC9A4 | solute carrier family 9 (sodium/hydrogen exchanger), member 4 | 1.13 | 0.00019 | 0.003 |
| 7934916 | CH25H | cholesterol 25-hydroxylase | 1.13 | 0.00009 | 0.002 |
| 7938055 | NA | ENSG00000181017 | 1.13 | 0.00071 | 0.008 |
| 8031157 | TTYH1 | tweety homolog 1 (Drosophila) | 1.13 | 0.00035 | 0.005 |
| 7940377 | TMEM132A | transmembrane protein 132A | 1.13 | 0.00059 | 0.007 |
| 7946031 | OR51V1 | olfactory receptor, family 51, subfamily V, member 1 | 1.13 | 0.00100 | 0.010 |
| 8026133 | DAND5 | DAN domain family, member 5 | 1.13 | 0.00041 | 0.005 |
| 8060101 | PP14571 | similar to hCG1777210 | 1.13 | 0.00081 | 0.008 |
| 8110347 | SLC34A1 | solute carrier family 34 (sodium phosphate), member 1 | 1.13 | 0.00022 | 0.004 |
| 8027556 | LRP3 | low density lipoprotein receptor-related protein 3 | 1.13 | 0.00073 | 0.008 |
| 8118890 | SCUBE3 | signal peptide, CUB domain, EGF-like 3 | 1.14 | 0.00006 | 0.002 |
| 7914000 | NR0B2 | nuclear receptor subfamily 0, group B, member 2 | 1.14 | 0.00010 | 0.002 |
| 8133500 | NA | Williams-Beuren syndrome chromosome region 23 | 1.14 | 0.00080 | 0.008 |
| 8100154 | CORIN | corin, serine peptidase | 1.14 | 0.00048 | 0.006 |
| 8120247 | NA |  | 1.14 | 0.00053 | 0.006 |
| 8015242 | KRTAP4-2 | keratin associated protein 4-2 | 1.14 | 0.00097 | 0.009 |
| 8008310 | EME1 | essential meiotic endonuclease 1 homolog 1 (S. pombe) | 1.14 | 0.00030 | 0.004 |
| 7945678 | NA | chromosome 11 open reading frame 89 | 1.14 | 0.00083 | 0.009 |
| 8141669 | C7orf52 | chromosome 7 open reading frame 52 | 1.14 | 0.00036 | 0.005 |
| 8087119 | SLC26A6 | solute carrier family 26, member 6; cadherin, EGF LAG seven-pass G-type receptor 3 (flamingo homolog, Drosophila) | 1.14 | 0.00101 | 0.010 |
| 8075529 | PISD | phosphatidylserine decarboxylase | 1.14 | 0.00060 | 0.007 |
| 8150352 | C8orf86 | chromosome 8 open reading frame 86 | 1.14 | 0.00011 | 0.002 |
| 8011324 | OR1G1 | olfactory receptor, family 1, subfamily G, member 1 | 1.14 | 0.00012 | 0.002 |
| 8109157 | NA | microRNA 143 | 1.14 | 0.00096 | 0.009 |
| 8136837 | OR6V1 | olfactory receptor, family 6, subfamily V, member 1 | 1.15 | 0.00011 | 0.002 |
| 8069811 | KRTAP23-1 | keratin associated protein 23-1 | 1.15 | 0.00047 | 0.006 |
| 7913566 | HTR1D | 5-hydroxytryptamine (serotonin) receptor 1D | 1.15 | 0.00040 | 0.005 |
| 7960397 | FGF23 | fibroblast growth factor 23 | 1.15 | 0.00003 | 0.001 |
| 8015240 | KRTAP4-3 | keratin associated protein 4-3 | 1.15 | 0.00052 | 0.006 |
| 7926368 | VIM | vimentin | 1.15 | 0.00083 | 0.009 |
| 7925759 | OR2T27 | olfactory receptor, family 2, subfamily T, member 7; olfactory receptor, family 2, subfamily T, member 27 | 1.16 | 0.00034 | 0.005 |
| 7988753 | SPPL2A | signal peptide peptidase-like 2A | 1.16 | 0.00023 | 0.004 |
| 8113616 | FEM1C | fem-1 homolog c (C. elegans) | 1.16 | 0.00020 | 0.003 |
| 7969796 | TM9SF2 | transmembrane 9 superfamily member 2 | 1.16 | 0.00057 | 0.007 |
| 8053364 | NA | ENSG00000208837 | 1.16 | 0.00019 | 0.003 |
| 8170468 | HMGB3 | similar to high mobility group box 3; high-mobility group box 3 | 1.16 | 0.00078 | 0.008 |
| 8010260 | BIRC5 | baculoviral IAP repeat-containing 5 | 1.16 | 0.00077 | 0.008 |
| 8001064 | VN1R3 | ENSG00000180663 | 1.16 | 0.00011 | 0.002 |
| 7923528 | MYOG | myogenin (myogenic factor 4) | 1.16 | 0.00004 | 0.001 |
| 7918913 | IGSF3 | immunoglobulin superfamily, member 3 | 1.17 | 0.00005 | 0.001 |
| 8139723 | FKBP9L | FK506 binding protein 9-like | 1.17 | 0.00015 | 0.003 |
| 8063028 | DNTTIP1 | deoxynucleotidyltransferase, terminal, interacting protein 1 | 1.17 | 0.00026 | 0.004 |
| 8094870 | SHISA3 | shisa homolog 3 (Xenopus laevis) | 1.17 | 0.00035 | 0.005 |
| 8102004 | NA | ENSG00000211265 | 1.18 | 0.00021 | 0.003 |
| 7914748 | NA |  | 1.18 | 0.00033 | 0.005 |
| 7937696 | KRTAP5-2 | hypothetical protein LOC338651 | 1.18 | 0.00022 | 0.003 |
| 8123760 | RP3-398D13.1 | hypothetical LOC285780 | 1.18 | 0.00049 | 0.006 |
| 7919301 | LOC100130236 | ENSG00000208912 | 1.18 | 0.00042 | 0.005 |
| 7967456 | RILPL2 | Rab interacting lysosomal protein-like 2 | 1.18 | 0.00095 | 0.009 |
| 7987012 | CHRFAM7A | CHRNA7 (cholinergic receptor, nicotinic, alpha 7, exons 5-10) and FAM7A (family with sequence similarity 7A, exons A-E) fusion; cholinergic receptor, nicotinic, alpha 7 | 1.19 | 0.00017 | 0.003 |
| 8116372 | RNF130 | ring finger protein 130 | 1.19 | 0.00008 | 0.002 |
| 8039086 | LOC284379 | solute carrier family 7 (cationic amino acid transporter, y+ system), member 3 pseudogene | 1.19 | 0.00001 | 0.000 |
| 8083569 | TIPARP | TCDD-inducible poly(ADP-ribose) polymerase | 1.19 | 0.00013 | 0.002 |
| 8000590 | SULT1A1 | sulfotransferase family, cytosolic, 1A, phenol-preferring, member 1 | 1.20 | 0.00073 | 0.008 |
| 7979473 | DHRS7 | dehydrogenase/reductase (SDR family) member 7 | 1.20 | 0.00040 | 0.005 |
| 7969414 | KLF5 | Kruppel-like factor 5 (intestinal) | 1.20 | 0.00079 | 0.008 |
| 8066619 | PLTP | phospholipid transfer protein | 1.21 | 0.00048 | 0.006 |
| 8180322 | KRTAP21-1 | keratin associated protein 21-1 | 1.22 | 0.00016 | 0.003 |
| 8122933 | TIAM2 | T-cell lymphoma invasion and metastasis 2 | 1.22 | 0.00016 | 0.003 |
| 7924758 | NA | hCG2040210 | 1.22 | 0.00052 | 0.006 |
| 8132290 | NA | S100 calcium binding protein A11; S100 calcium binding protein A11 pseudogene | 1.22 | 0.00002 | 0.001 |
| 7981775 | NA | hypothetical protein DKFZp547L112 | 1.22 | 0.00001 | 0.000 |
| 8073960 | PIM3 | pim-3 oncogene | 1.23 | 0.00038 | 0.005 |
| 8121563 | MARCKS | myristoylated alanine-rich protein kinase C substrate | 1.24 | 0.00015 | 0.003 |
| 7896391 | NA |  | 1.25 | 0.00077 | 0.008 |
| 8131871 | CCDC126 | coiled-coil domain containing 126 | 1.25 | 0.00079 | 0.008 |
| 7896051 | NA |  | 1.27 | 0.00011 | 0.002 |
| 8107706 | LMNB1 | lamin B1 | 1.28 | 0.00023 | 0.004 |
| 8151711 | NBN | nibrin | 1.28 | 0.00064 | 0.007 |
| 8133027 | LOC100132217 | ENSG00000188185 | 1.28 | 0.00019 | 0.003 |
| 7895450 | NA |  | 1.32 | 0.00056 | 0.007 |
| 7892878 | NA |  | 1.39 | 0.00043 | 0.006 |
| 8077366 | LRRN1 | leucine rich repeat neuronal 1 | 1.49 | 0.00046 | 0.006 |
| 7895713 | NA |  | 1.68 | 0.00055 | 0.007 |

Table S7. Alteration of the cell cycle pathway (5^th^ day of life)

Genes up-regulated

| gene abreviation | gene name | Fold change | adjusted p value |
| --- | --- | --- | --- |
| BUB1 | budding uninhibited by benzimidazoles 1 homolog (yeast) | 1.2811353 | 0.0081706 |
| BUB1B | budding uninhibited by benzimidazoles 1 homolog beta (yeast) | 1.5578689 | 0.0001423 |
| CCNA2 | cyclin A2 | 1.5037063 | 0.000438 |
| CCNB2 | cyclin B2 | 1.390677 | 0.0109699 |
| CCNE2 | cyclin E2 | 1.5594559 | 0.0019168 |
| CDC25A | cell division cycle 25 homolog A (S. pombe) | 1.3963371 | 8.456E-05 |
| CDC25C | cell division cycle 25 homolog C (S. pombe) | 1.1810961 | 0.0023979 |
| CDC45 | cell division cycle 45 homolog (S. cerevisiae) | 1.3077524 | 0.0016458 |
| CDC6 | cell division cycle 6 homolog (S. cerevisiae) | 1.5535671 | 0.0005534 |
| CDK1 | cyclin-dependent kinase 1 | 1.5268368 | 0.0039899 |
| CHEK1 | CHK1 checkpoint homolog (S. pombe) | 1.3177526 | 0.0091217 |
| E2F3 | E2F transcription factor 3 | 1.2673455 | 0.0075573 |
| ESPL1 | extra spindle pole bodies homolog 1 (S. cerevisiae) | 1.3072388 | 0.0001206 |
| GADD45A | growth arrest and DNA-damage-inducible, alpha | 1.5189563 | 4.352E-05 |
| GADD45G | growth arrest and DNA-damage-inducible, gamma | 1.1879208 | 0.0021533 |
| MCM2 | minichromosome maintenance complex component 2 | 1.1749774 | 0.045838 |
| MCM4 | minichromosome maintenance complex component 4 | 1.2863305 | 0.0042561 |
| ORC1 | origin recognition complex, subunit 1 | 1.4345764 | 0.0001175 |
| ORC6 | origin recognition complex, subunit 6 | 1.2643345 | 0.0017163 |
| PCNA | proliferating cell nuclear antigen | 1.279917 | 0.0082624 |
| PKMYT1 | protein kinase, membrane associated tyrosine | 1.2143276 | 0.0005551 |
| PLK1 | polo-like kinase 1 | 1.2497388 | 0.0174394 |
| PTTG2 | pituitary tumor-transforming 2 | 1.4227765 | 0.0283692 |
| TTK | TTK protein kinase | 1.446117 | 0.0024113 |
| WEE1 | WEE1 homolog (S. pombe) | 1.3645572 | 0.0035376 |
| YWHAH | tyrosine 3-monooxygenase | 1.1777165 | 0.0322726 |

Genes down-regulated

| TP53 | tumor protein p53 | 0.7869784 | 0.0004684 |
| --- | --- | --- | --- |
| ORC5 | origin recognition complex, subunit 5 | 0.8488186 | 0.0028463 |
| MYC | v-myc myelocytomatosis viral oncogene homolog (avian) | 0.7887987 | 0.003732 |
| CDC25B | cell division cycle 25 homolog B (S. pombe) | 0.7606452 | 0.0050768 |
| TGFB2 | transforming growth factor, beta 2 | 0.8773138 | 0.0067508 |
| CDC16 | cell division cycle 16 homolog (S. cerevisiae) | 0.8794711 | 0.0155394 |
| ANAPC5 | anaphase promoting complex subunit 5 | 0.8937349 | 0.0428372 |

Genes not altered

| ABL1 | c-abl oncogene 1, non-receptor tyrosine kinase | 0.9874645 | 0.8216166 |
| --- | --- | --- | --- |
| ANAPC1 | anaphase promoting complex subunit 1 | 0.8850575 | 0.1166716 |
| ANAPC10 | anaphase promoting complex subunit 10 | 0.9846841 | 0.8741919 |
| ANAPC11 | anaphase promoting complex subunit 11 | 0.9716212 | 0.7166668 |
| ANAPC13 | anaphase promoting complex subunit 13 | 0.927215 | 0.2004577 |
| ANAPC2 | anaphase promoting complex subunit 2 | 0.9286301 | 0.0976561 |
| ANAPC4 | anaphase promoting complex subunit 4 | 1.0462595 | 0.6455572 |
| ANAPC7 | anaphase promoting complex subunit 7 | 0.9976485 | 0.966048 |
| ATM | ataxia telangiectasia mutated | 0.8116902 | 0.0646163 |
| ATR | ataxia telangiectasia and Rad3 related | 0.9002031 | 0.361944 |
| BUB3 | budding uninhibited by benzimidazoles 3 homolog (yeast) | 0.8936255 | 0.0737976 |
| CCNA1 | cyclin A1 | 1.0391979 | 0.5856056 |
| CCNB1 | cyclin B1 | 1.129222 | 0.2737 |
| CCNB3 | cyclin B3 | 0.9796269 | 0.6903015 |
| CCND1 | cyclin D1 | 1.0688011 | 0.1093063 |
| CCND2 | cyclin D2 | 0.9266619 | 0.2929546 |
| CCND3 | cyclin D3 | 1.1382056 | 0.1057921 |
| CCNE1 | cyclin E1 | 1.0553439 | 0.3991842 |
| CCNH | cyclin H | 1.0486982 | 0.5968971 |
| CDC14A | CDC14 cell division cycle 14 homolog A (S. cerevisiae) | 0.932167 | 0.5352358 |
| CDC14B | CDC14 cell division cycle 14 homolog B (S. cerevisiae) | 0.8601728 | 0.2535151 |
| CDC20 | cell division cycle 20 homolog (S. cerevisiae) | 1.1485155 | 0.1083876 |
| CDC23 | cell division cycle 23 homolog (S. cerevisiae) | 0.9193326 | 0.2582969 |
| CDC26 | cell division cycle 26 homolog (S. cerevisiae) | 0.9462006 | 0.5923204 |
| CDC27 | cell division cycle 27 homolog (S. cerevisiae) | 1.0121078 | 0.8899033 |
| CDC7 | cell division cycle 7 homolog (S. cerevisiae) | 1.1689182 | 0.1154253 |
| CDK2 | cyclin-dependent kinase 2 | 1.1296569 | 0.0880691 |
| CDK4 | cyclin-dependent kinase 4 | 0.9418211 | 0.5317985 |
| CDK6 | cyclin-dependent kinase 6 | 1.0820291 | 0.4490293 |
| CDK7 | cyclin-dependent kinase 7 | 1.0534449 | 0.4949399 |
| CDKN1A | cyclin-dependent kinase inhibitor 1A (p21, Cip1) | 0.8882306 | 0.2130976 |
| CDKN1B | cyclin-dependent kinase inhibitor 1B (p27, Kip1) | 1.016271 | 0.8096554 |
| CDKN1C | cyclin-dependent kinase inhibitor 1C (p57, Kip2) | 1.0713779 | 0.2977188 |
| CDKN2A | cyclin-dependent kinase inhibitor 2A (melanoma, p16, inhibits CDK4) | 0.9571976 | 0.5826215 |
| CDKN2B | cyclin-dependent kinase inhibitor 2B (p15, inhibits CDK4) | 0.9560675 | 0.5517568 |
| CDKN2C | cyclin-dependent kinase inhibitor 2C (p18, inhibits CDK4) | 1.0246261 | 0.7124564 |
| CDKN2D | cyclin-dependent kinase inhibitor 2D (p19, inhibits CDK4) | 1.1056221 | 0.1627048 |
| CHEK2 | CHK2 checkpoint homolog (S. pombe) | 0.9189072 | 0.1262357 |
| CREBBP | CREB binding protein | 1.1128471 | 0.2234799 |
| CUL1 | cullin 1 | 1.0040337 | 0.957658 |
| DBF4 | DBF4 homolog (S. cerevisiae) | 0.9859076 | 0.8812763 |
| E2F1 | E2F transcription factor 1 | 0.9675648 | 0.6980194 |
| E2F2 | E2F transcription factor 2 | 1.183444 | 0.1177736 |
| E2F4 | E2F transcription factor 4, p107 | 0.9307211 | 0.1971856 |
| E2F5 | E2F transcription factor 5, p130-binding | 0.870242 | 0.3090135 |
| EP300 | E1A binding protein p300 | 1.1711328 | 0.1021825 |
| FZR1 | Fizzy | 1.0064735 | 0.921329 |
| GADD45B | growth arrest and DNA-damage-inducible, beta | 1.0517023 | 0.5249517 |
| GSK3B | glycogen synthase kinase 3 beta | 1.1095274 | 0.1848965 |
| HDAC1 | histone deacetylase 1 | 0.8918662 | 0.0733245 |
| HDAC2 | histone deacetylase 2 | 0.9742215 | 0.6969708 |
| MAD1L1 | MAD1 mitotic arrest deficient-like 1 (yeast) | 0.9687922 | 0.3633922 |
| MAD2L1 | MAD2 mitotic arrest deficient-like 1 (yeast) | 1.1355244 | 0.17602 |
| MAD2L2 | MAD2 mitotic arrest deficient-like 2 (yeast) | 0.9710549 | 0.7180086 |
| MCM3 | minichromosome maintenance complex component 3 | 1.0595987 | 0.5222413 |
| MCM5 | minichromosome maintenance complex component 5 | 1.0500945 | 0.589887 |
| MCM6 | minichromosome maintenance complex component 6 | 1.0801701 | 0.4795227 |
| MCM7 | minichromosome maintenance complex component 7 | 1.0693276 | 0.4316195 |
| MDM2 | Mdm2 p53 binding protein homolog (mouse) | 1.045254 | 0.5107883 |
| ORC2 | origin recognition complex, subunit 2 | 0.9756728 | 0.7018065 |
| ORC3 | origin recognition complex, subunit 3 | 0.9626853 | 0.6548221 |
| ORC4 | origin recognition complex, subunit 4 | 0.9619058 | 0.5738677 |
| PRKDC | protein kinase, DNA-activated, catalytic polypeptide | 1.0857416 | 0.499516 |
| PTTG1 | pituitary tumor-transforming 1 | 1.2126375 | 0.0570335 |
| RAD21 | RAD21 homolog (S. pombe) | 1.0631475 | 0.2513107 |
| RB1 | retinoblastoma 1 | 1.0025046 | 0.9810074 |
| RBL1 | retinoblastoma-like 1 (p107) | 1.117368 | 0.1639997 |
| RBL2 | retinoblastoma-like 2 (p130) | 0.8917769 | 0.0771169 |
| RBX1 | ring-box 1, E3 ubiquitin protein ligase | 0.9554219 | 0.6044002 |
| SFN | Stratifin | 1.0495853 | 0.3450185 |
| SKP1 | S-phase kinase-associated protein 1 | 1.0484578 | 0.577371 |
| SKP2 | S-phase kinase-associated protein 2 (p45) | 1.1023549 | 0.1931302 |
| SMAD2 | SMAD family member 2 | 1.1586882 | 0.2700385 |
| SMAD3 | SMAD family member 3 | 0.9318264 | 0.3624975 |
| SMAD4 | SMAD family member 4 | 0.9968743 | 0.9627736 |
| SMC1A | structural maintenance of chromosomes 1A | 0.9657396 | 0.6869691 |
| SMC1B | structural maintenance of chromosomes 1B | 0.9957553 | 0.9457304 |
| SMC3 | structural maintenance of chromosomes 3 | 1.0971167 | 0.2094855 |
| STAG1 | stromal antigen 1 | 1.0609881 | 0.5238972 |
| STAG2 | stromal antigen 2 | 1.0558822 | 0.4587928 |
| TFDP1 | transcription factor Dp-1 | 1.1416083 | 0.065364 |
| TFDP2 | transcription factor Dp-2 (E2F dimerization partner 2) | 0.9763268 | 0.8258491 |
| TGFB1 | transforming growth factor, beta 1 | 0.9354921 | 0.4523116 |
| TGFB3 | transforming growth factor, beta 3 | 0.9691352 | 0.5463963 |
| YWHAB | tyrosine 3-monooxygenase | 0.9882288 | 0.8440278 |
| YWHAE | tyrosine 3-monooxygenase | 0.9202975 | 0.2741257 |
| YWHAG | tyrosine 3-monooxygenase | 0.9669229 | 0.5992932 |
| YWHAQ | tyrosine 3-monooxygenase | 0.9228705 | 0.2709838 |
| YWHAZ | tyrosine 3-monooxygenase | 1.006043 | 0.9214981 |
| ZBTB17 | zinc finger and BTB domain containing 17 | 1.0090239 | 0.84415 |

Table S8. Alteration of the T cell receptor signaling pathway (5^th^ day of life)

**Down-regulated genes**

|  | Gene name | Fold change | Adjusted p value |
| --- | --- | --- | --- |
| AKT2 | v-akt murine thymoma viral oncogene homolog 2 | 0.9148909 | 0.0269275 |
| AKT3 | v-akt murine thymoma viral oncogene homolog 3 (protein kinase B, gamma) | 0.687449 | 2.6E-05 |
| CARD11 | caspase recruitment domain family, member 11 | 0.7767871 | 0.002423 |
| CD247 | CD247 molecule | 0.6382669 | 0.0001129 |
| CD28 | CD28 molecule | 0.5759957 | 2.863E-05 |
| CD3D | CD3d molecule, delta (CD3-TCR complex) | 0.5495185 | 6.734E-05 |
| CD3E | CD3e molecule, epsilon (CD3-TCR complex) | 0.5991271 | 3.576E-05 |
| CD3G | CD3g molecule, gamma (CD3-TCR complex) | 0.5344595 | 2.233E-05 |
| CD4 | CD4 molecule | 0.6280471 | 2.123E-05 |
| CD40LG | CD40 ligand | 0.6498562 | 3.139E-05 |
| CD8A | CD8a molecule | 0.7085937 | 0.0014069 |
| CD8B | CD8b molecule | 0.682406 | 0.0001812 |
| FYN | FYN oncogene related to SRC, FGR, YES | 0.8028087 | 0.0102714 |
| GRAP2 | GRB2-related adaptor protein 2 | 0.6865192 | 0.0073058 |
| ICOS | inducible T-cell co-stimulator | 0.5628876 | 2.844E-05 |
| IFNG | interferon, gamma | 0.8697518 | 0.0294745 |
| IKBKB | inhibitor of kappa light polypeptide gene enhancer in B-cells, kinase beta | 0.8982598 | 0.0113295 |
| ITK | IL2-inducible T-cell kinase | 0.5746002 | 8.502E-05 |
| LAT | linker for activation of T cells | 0.8154444 | 0.0349313 |
| LCK | lymphocyte-specific protein tyrosine kinase | 0.671136 | 0.0007889 |
| MALT1 | mucosa associated lymphoid tissue lymphoma translocation gene 1 | 0.8124011 | 0.0016831 |
| MAP3K14 | mitogen-activated protein kinase kinase kinase 14 | 0.8758822 | 0.0047161 |
| NFATC2 | nuclear factor of activated T-cells, cytoplasmic, calcineurin-dependent 2 | 0.7895215 | 0.0140152 |
| NFKBIE | nuclear factor of kappa light polypeptide gene enhancer in B-cells inhibitor, epsilon | 0.8782707 | 0.0283083 |
| PIK3R2 | phosphoinositide-3-kinase, regulatory subunit 2 (beta) | 0.8849808 | 0.0398685 |
| PIK3R3 | phosphoinositide-3-kinase, regulatory subunit 3 (gamma) | 0.8000036 | 0.0010557 |
| PLCG1 | phospholipase C, gamma 1 | 0.6514217 | 0.0003293 |
| PPP3CC | protein phosphatase 3, catalytic subunit, gamma isozyme | 0.8217376 | 0.0062815 |
| PRKCQ | protein kinase C, theta | 0.7897469 | 0.0159223 |
| RASGRP1 | RAS guanyl releasing protein 1 (calcium and DAG-regulated) | 0.6991805 | 0.0004712 |
| RELA | v-rel reticuloendotheliosis viral oncogene homolog A (avian) | 0.92032 | 0.0371152 |
| ZAP70 | zeta-chain (TCR) associated protein kinase 70kDa | 0.6701715 | 0.000495 |

**Up-regulated genes**

|  | Gene name | Fold change | Adjusted p value |
| --- | --- | --- | --- |
| MAP3K8 | mitogen-activated protein kinase kinase kinase 8 | 1.2151043 | 0.0011455 |
| MAPK1 | mitogen-activated protein kinase 1 | 1.118641 | 0.0239827 |
| MAPK12 | mitogen-activated protein kinase 12 | 1.1035109 | 0.0174971 |
| MAPK14 | mitogen-activated protein kinase 14 | 1.3428947 | 0.0007271 |
| PIK3CB | phosphoinositide-3-kinase, catalytic, beta polypeptide | 1.2545698 | 0.015742 |
| PIK3CG | phosphoinositide-3-kinase, catalytic, gamma polypeptide | 1.2279518 | 0.0079865 |
| RAF1 | v-raf-1 murine leukemia viral oncogene homolog 1 | 1.1518003 | 0.023829 |
| SOS2 | son of sevenless homolog 2 (Drosophila) | 1.3162165 | 0.0091153 |
| TEC | tec protein tyrosine kinase | 1.3951261 | 0.0008155 |
| VAV3 | vav 3 guanine nucleotide exchange factor | 1.2735117 | 0.0256119 |

**Genes not alterred**

|  | Gene name | Fold change | Adjusted p value |
| --- | --- | --- | --- |
| AKT1 | v-akt murine thymoma viral oncogene homolog 1 | 0.8952881 | 0.0669845 |
| BCL10 | B-cell CLL | 1.0178519 | 0.8006506 |
| CBL | Cas-Br-M (murine) ecotropic retroviral transforming sequence | 1.1526558 | 0.1179342 |
| CBLB | Cas-Br-M (murine) ecotropic retroviral transforming sequence b | 0.8390424 | 0.1195227 |
| CBLC | Cas-Br-M (murine) ecotropic retroviral transforming sequence c | 1.0443531 | 0.4258847 |
| CDC42 | cell division cycle 42 (GTP binding protein, 25kDa) | 1.0010688 | 0.9901895 |
| CDK4 | cyclin-dependent kinase 4 | 0.9418211 | 0.5317985 |
| CHP | calcium binding protein P22 | 0.9441198 | 0.3909204 |
| CHP2 | calcineurin B homologous protein 2 | 1.0594725 | 0.3467343 |
| CHUK | conserved helix-loop-helix ubiquitous kinase | 1.1252644 | 0.1360851 |
| CSF2 | colony stimulating factor 2 (granulocyte-macrophage) | 0.9660181 | 0.4444351 |
| CTLA4 | cytotoxic T-lymphocyte-associated protein 4 | 0.8745004 | 0.5086712 |
| DLG1 | discs, large homolog 1 (Drosophila) | 0.9969568 | 0.9803811 |
| FOS | FBJ murine osteosarcoma viral oncogene homolog | 1.0720022 | 0.7188096 |
| GRB2 | growth factor receptor-bound protein 2 | 0.9988462 | 0.9844406 |
| GSK3B | glycogen synthase kinase 3 beta | 1.1095274 | 0.1848965 |
| HRAS | v-Ha-ras Harvey rat sarcoma viral oncogene homolog | 0.8975934 | 0.0631961 |
| IKBKG | inhibitor of kappa light polypeptide gene enhancer in B-cells, kinase gamma | 0.9583489 | 0.6319867 |
| IL10 | interleukin 10 | 1.0203225 | 0.828446 |
| IL2 | interleukin 2 | 0.9689734 | 0.4433182 |
| IL4 | interleukin 4 | 0.9848812 | 0.7568129 |
| IL5 | interleukin 5 (colony-stimulating factor, eosinophil) | 0.9984581 | 0.9807563 |
| JUN | jun proto-oncogene | 1.0324644 | 0.902102 |
| KRAS | v-Ki-ras2 Kirsten rat sarcoma viral oncogene homolog | 1.0981141 | 0.21347 |
| LCP2 | lymphocyte cytosolic protein 2 (SH2 domain containing leukocyte protein of 76kDa) | 1.0359736 | 0.5242002 |
| MAP2K1 | mitogen-activated protein kinase kinase 1 | 1.0118819 | 0.8882487 |
| MAP2K2 | mitogen-activated protein kinase kinase 2 | 1.0472959 | 0.5835691 |
| MAP2K7 | mitogen-activated protein kinase kinase 7 | 0.9178622 | 0.0577407 |
| MAP3K7 | mitogen-activated protein kinase kinase kinase 7 | 1.023232 | 0.7708455 |
| MAPK11 | mitogen-activated protein kinase 11 | 1.0092338 | 0.8995269 |
| MAPK13 | mitogen-activated protein kinase 13 | 1.1145971 | 0.1462387 |
| MAPK3 | mitogen-activated protein kinase 3 | 1.0093367 | 0.9274912 |
| MAPK9 | mitogen-activated protein kinase 9 | 1.0612498 | 0.4630228 |
| NA | protein phosphatase 3, regulatory subunit B, alpha | 1.054744 | 0.3900091 |
| NCK1 | NCK adaptor protein 1 | 0.9334461 | 0.2794147 |
| NCK2 | NCK adaptor protein 2 | 0.9304492 | 0.3173978 |
| NFAT5 | nuclear factor of activated T-cells 5, tonicity-responsive | 1.1448381 | 0.2840666 |
| NFATC1 | nuclear factor of activated T-cells, cytoplasmic, calcineurin-dependent 1 | 0.9053669 | 0.0704396 |
| NFATC3 | nuclear factor of activated T-cells, cytoplasmic, calcineurin-dependent 3 | 0.9377628 | 0.3352115 |
| NFATC4 | nuclear factor of activated T-cells, cytoplasmic, calcineurin-dependent 4 | 1.0309481 | 0.585354 |
| NFKB1 | nuclear factor of kappa light polypeptide gene enhancer in B-cells 1 | 0.9604357 | 0.5245278 |
| NFKBIA | nuclear factor of kappa light polypeptide gene enhancer in B-cells inhibitor, alpha | 1.1495035 | 0.2491674 |
| NFKBIB | nuclear factor of kappa light polypeptide gene enhancer in B-cells inhibitor, beta | 0.9784037 | 0.6958828 |
| NRAS | neuroblastoma RAS viral (v-ras) oncogene homolog | 0.9207036 | 0.1004509 |
| PAK1 | p21 protein (Cdc42 | 1.0637625 | 0.3727429 |
| PAK2 | p21 protein (Cdc42 | 1.100423 | 0.1828592 |
| PAK3 | p21 protein (Cdc42 | 0.9953366 | 0.9403415 |
| PAK4 | p21 protein (Cdc42 | 0.9699027 | 0.4838726 |
| PAK6 | p21 protein (Cdc42 | 1.0214079 | 0.7008522 |
| PAK7 | p21 protein (Cdc42 | 0.9765707 | 0.6128838 |
| PDCD1 | programmed cell death 1 | 1.020634 | 0.7177888 |
| PDK1 | pyruvate dehydrogenase kinase, isozyme 1 | 0.9197437 | 0.3287601 |
| PIK3CA | phosphoinositide-3-kinase, catalytic, alpha polypeptide | 1.0358236 | 0.6945547 |
| PIK3CD | phosphoinositide-3-kinase, catalytic, delta polypeptide | 0.9800618 | 0.7557735 |
| PIK3R1 | phosphoinositide-3-kinase, regulatory subunit 1 (alpha) | 0.9959692 | 0.9578283 |
| PIK3R5 | phosphoinositide-3-kinase, regulatory subunit 5 | 1.0173911 | 0.8263042 |
| PPP3CA | protein phosphatase 3, catalytic subunit, alpha isozyme | 1.1478102 | 0.0509936 |
| PPP3CB | protein phosphatase 3, catalytic subunit, beta isozyme | 1.0909211 | 0.2018587 |
| PPP3R2 | protein phosphatase 3, regulatory subunit B, beta | 1.0503501 | 0.5409232 |
| PTPN6 | protein tyrosine phosphatase, non-receptor type 6 | 1.041334 | 0.4824775 |
| PTPRC | protein tyrosine phosphatase, receptor type, C | 0.9947025 | 0.9495759 |
| RHOA | ras homolog gene family, member A | 1.070119 | 0.0866332 |
| SOS1 | son of sevenless homolog 1 (Drosophila) | 1.1299149 | 0.3316513 |
| TNF | tumor necrosis factor | 1.0229457 | 0.8125774 |
| VAV1 | vav 1 guanine nucleotide exchange factor | 1.0126837 | 0.843087 |
| VAV2 | vav 2 guanine nucleotide exchange factor | 0.8521568 | 0.0558001 |

Figure S8. Heatmap from clustering analysis of genes with known gene symbol. Genes are clustered by similarity in gene expression values (log2 values) on the 5^th^ day of life. The columns indicated in grey represent the patients without BPD, whereas the columns indicated in black represent the patients with BPD.


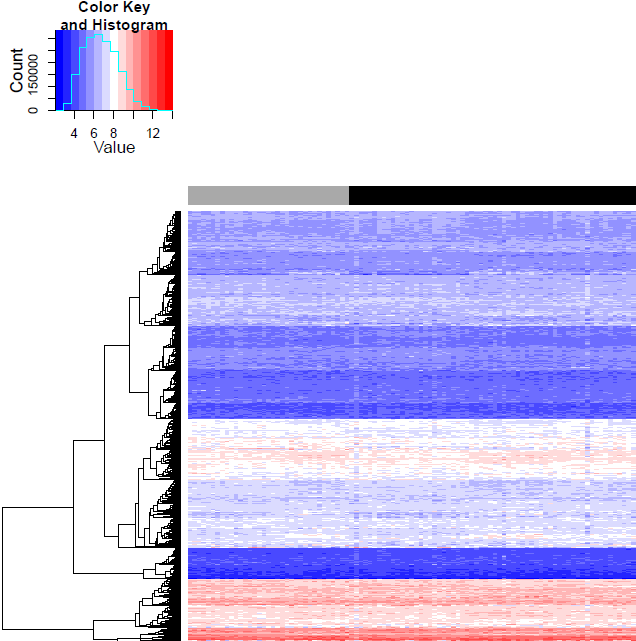


Figure S9. Heatmap from clustering analysis of genes with known gene symbol. Genes are clustered by similarity in gene expression values (log2 values) on the 14^th^ day of life. The columns indicated in grey represent the patients without BPD, whereas the columns indicated in black represent the patients with BPD.


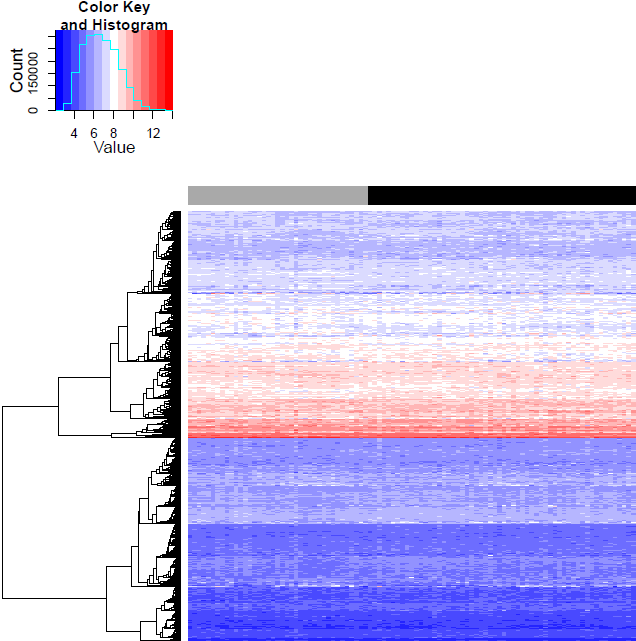


Figure S10. Heatmap from clustering analysis of genes with known gene symbol. Genes are clustered by similarity in gene expression values (log2 values) on the 28^th^ day of life. The columns indicated in grey represent the patients without BPD, whereas the columns indicated in black represent the patients with BPD.


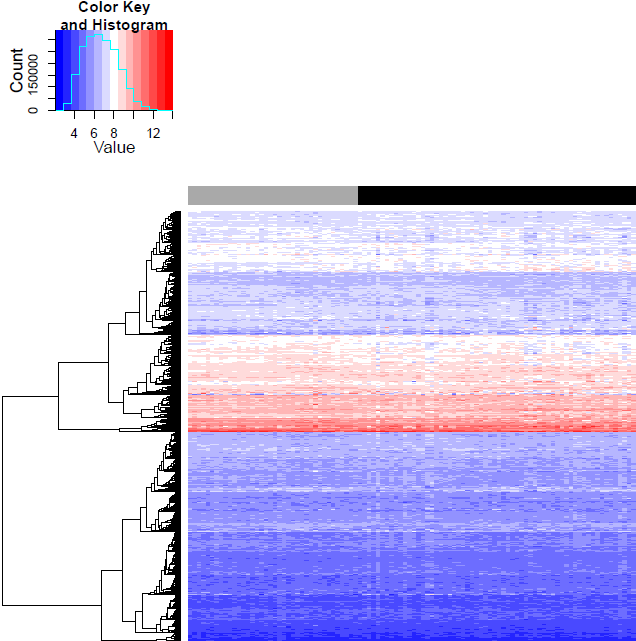

Supplement: Appendix S1 — Detailed description of microarray evaluation, tables S1-S8, figures S1-S10. (DOCX) [file pone.0078585.s001.docx]
